# Relevance of MicroRNAs as Potential Diagnostic and Prognostic Markers in Colorectal Cancer

**DOI:** 10.3390/ijms19102944

**Published:** 2018-09-27

**Authors:** Grzegorz Hibner, Małgorzata Kimsa-Furdzik, Tomasz Francuz

**Affiliations:** Department of Biochemistry, School of Medicine in Katowice, Medical University of Silesia in Katowice, St. Medyków 18, 40-752 Katowice, Poland; malgorzata.kimsa@sum.edu.pl (M.K.-F.); tfrancuz@sum.edu.pl (T.F.)

**Keywords:** colorectal, cancer, microRNA, biomarkers

## Abstract

Colorectal cancer (CRC) is currently the third and the second most common cancer in men and in women, respectively. Every year, more than one million new CRC cases and more than half a million deaths are reported worldwide. The majority of new cases occur in developed countries. Current screening methods have significant limitations. Therefore, a lot of scientific effort is put into the development of new diagnostic biomarkers of CRC. Currently used prognostic markers are also limited in assessing the effectiveness of CRC therapy. MicroRNAs (miRNAs) are a promising subject of research especially since single miRNA can recognize a variety of different mRNA transcripts. MiRNAs have important roles in epigenetic regulation of basic cellular processes, such as proliferation, apoptosis, differentiation, and migration, and may serve as potential oncogenes or tumor suppressors during cancer development. Indeed, in a large variety of human tumors, including CRC, significant distortions in miRNA expression profiles have been observed. Thus, the use of miRNAs as diagnostic and prognostic biomarkers in cancer, particularly in CRC, appears to be an inevitable consequence of the advancement in oncology and gastroenterology. Here, we review the literature to discuss the potential usefulness of selected miRNAs as diagnostic and prognostic biomarkers in CRC.

## 1. Introduction

Colorectal cancer (CRC) accounts for about 10% of all cancer cases worldwide. The latest Global Cancer Observatory data from 2012 estimates that nearly 1.4 million new cases of CRC and over 694,000 deaths were reported. CRC is more common in developed countries, with over 65% of cases. In Europe, about 400,000 new patients are diagnosed with CRC each year, and more than 200,000 die annually. The majority of CRCs occur in patients over the age of 50, with more than 75% being those over 60 years old. The risk of CRC increases with age and is 1.5–2 times higher in men than in women. Over the past three decades, in the male population, there has been a steady increase in mortality. In the female population, the increase in mortality stabilized in the mid-1990s and since then, mortality has remained relatively constant [1]. Currently used screening methods, including the fecal occult blood test (FOBT), stool DNA test, double-contrast barium enema (DCBE), and colonoscopy, have significant limitations. Colonoscopy is an invasive procedure that carries the risk of bowel perforation. Additionally, due to the nature of this test, some patients deny its implementation. Furthermore, the other tests mentioned are limited by either insufficient sensitivity or high percentage of false positive results. Therefore, currently, the development of new biomarkers for CRC screening tests is the subject of intensive research. However, most of the results of recent studies require confirmation on larger groups of patients before being implemented in clinical practice [2].

Currently, tumor-node-metastasis (TNM) classification is the primary prognostic marker in CRC therapy. The TNM system describes the size of the primary tumor, the degree of invasion of the intestinal wall, nearby lymph nodes and distant organs [3]. Although the TNM classification is the basis for CRC prognosis, this system has important caveats. Insufficient analysis of lymph node status may lead to underestimation of tumor progression, which in turn may result in ineffective treatment [3]. In addition, histologically indistinguishable CRCs may have various genetic and epigenetic backgrounds that contribute to different progressions and responses to treatment. For example, patients with TNM stage II CRC without lymph node metastasis have relatively good survival rates, but still around 25% of these patients have a high risk of relapse after surgical removal of the tumor. Unfortunately, currently used CRC prognostic markers have limited use in identification of patients with increased risk of recurrence and are still not highly accurate in assessing effectiveness of treatment.

**Figure 1:**
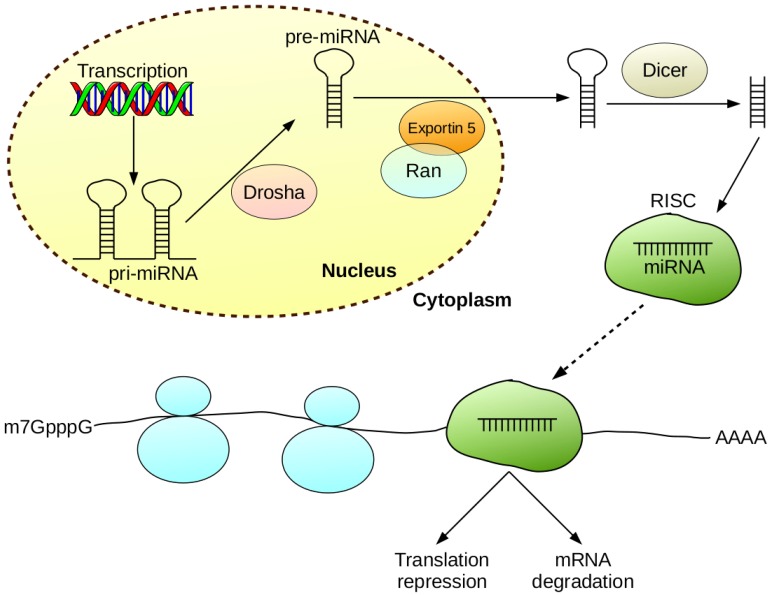
Synthesis and mechanism of miRNA activity.

MicroRNAs (miRNAs), which are short, single-stranded non-coding RNA sequences of approximately 21–23 nucleotides, are interesting and promising targets in CRC therapy and diagnostics. MiRNAs are products of intracellular processing of hairpin precursor molecules. Firstly, a miRNA gene is transcribed into a primary miRNA (pri-miRNA), which is then processed in the nucleus by Drosha RNase to form a precursor miRNA (pre-miRNA). The pre-miRNA is then transported to the cytoplasm via the activity of the exportin 5, which interacts with the Ran protein. In the cytoplasm, Dicer RNase is responsible for further processing of pre-miRNA. Subsequently, mature miRNA is complexed with Argonaute family proteins to form the RISC complex. Physiologically, miRNAs are responsible for epigenetic regulation of translation by attaching to the 3′-untranslated region (3′-UTR) of the target messenger RNA (mRNA) (Figure 1). In this way, miRNA mediates mRNA degradation and the repression of translation [4].

One type of miRNA molecules can regulate the expression of multiple target genes and activation of different signaling pathways, and their crosstalk. The miRNA expression is unique for different tissues (including tumor tissue), and it participates in maintaining a differentiated cellular state. Therefore, disorders of miRNA expression may shift the cell to the undifferentiated proliferative phenotype. MiRNA expression disorders may be caused by a variety of molecular processes, such as deletions, amplifications, mutations within the miRNA *locus*, epigenetic silencing, or abnormal regulation of transcription factor activity. It is estimated that miRNAs are responsible for the regulation of translation of about 30–60% of human genes [5,6]. Because miRNAs recognize many different mRNA transcripts, these molecules are important in epigenetic regulation of basic cell processes, such as proliferation, apoptosis, differentiation, and migration. Therefore, miRNAs may act as potential oncogenes or suppressors in tumor development processes. Indeed, for many different human cancers, including CRC, significant abnormalities in miRNA expression have been observed. In CRCs, the altered expression of a large number of miRNAs associated with the development, progression and response to the treatment of this cancer was observed [7,8,9]. Changes in miRNA expression are associated with more frequent metastases, promotion of tumor mass growth, and increased malignancy of tumor cells. MiRNA expression is also associated with the risk of recurrence, response to the therapeutic regimen, and survival time in different cancers. Therefore, miRNA profiling may be a new and valuable tool in the diagnosis and prognosis of many types of cancer, including CRC. However, knowledge in this area still remains fragmented, and previous studies were conducted only on small groups of patients. In addition, the vast majority of studies conducted so far have evaluated the expression of miRNAs only in tumor tissues. However, miRNA expression profiling in tumor tissue has significant disadvantages. The heterogeneity of tumor cells produces variable results. Obtaining reliable data in this case is problematic and requires the isolation of individual cancer cells, e.g., using the laser microdissection method. This method, however, is costly, requires specialized equipment and is therefore not commonly used in clinical practice. Furthermore, studying tumor tissues does not allow for the assessment of changes in miRNA expression during anti-cancer therapy. Alternatively, choosing serum or blood plasma as the test material is a more practical and cheaper approach to miRNA profiling. Stool is also suitable for miRNA expression analysis. In both cases, the material is readily available, which makes it possible to identify potential biomarkers at any stage during and after the therapy, e.g., for early detection of cancer recurrence. MiRNA in plasma and serum is encapsulated in exosomes, which makes it highly stable. Therefore, the material does not require any special storage or protection operations prior to analysis. Potential applications of miRNA expression analysis for diagnostic, therapeutic and prognostic purposes are summarized in Table 1. The use of miRNAs as diagnostic and prognostic markers in neoplastic diseases, particularly in CRC, appears to be an inevitable consequence of advances in clinical oncology and gastroenterology.

## 2. MiRNAs as Diagnostic Biomarkers

Circulating miRNAs are stable due to encapsulation in exosomes [10], but the mechanism of their formation and secretion has not yet been fully understood. There is increasing evidence that miRNAs contained in exosomes may be involved in the exchange of information between distant tissues [11]. Serum miRNA expression is known to be altered in CRC patients and current studies attempt to investigate the correlation of the expression of certain types of miRNAs, both in serum and tumor tissue. Due to minimal invasiveness of miRNA harvesting procedures, circulating miRNAs may be potentially used as diagnostic biomarkers of various cancers, including CRC. A significant limitation of miRNA expression analysis is its poor utility in diagnosis, due to non-specificity and high variability of expression of a single miRNA type. Therefore, recent studies have attempted to evaluate the expression of miRNA sets. Such studies were conducted using microarray technology as well as quantitative real-time reverse transcriptase polymerase chain reaction (qRT-PCR) [12]. Insufficient sensitivity resulting from low concentrations of miRNA in serum or plasma of the patients is the principal limitation of a microarray experiment. QRT-PCR is characterized by better sensitivity, but evaluation of expression of numerous miRNAs using this method is a difficult and time-consuming task. Next-generation sequencing (NGS) is a novel and promising method that may be applied to evaluate the expression profiles of many miRNAs simultaneously [13]. Moreover, novel isolation techniques allow obtaining more miRNA for analysis, which, in combination with specialized NGS protocols, can increase the utility of this method in diagnostics of cancer, including CRC in the near future.

Some non-invasive screening methods used in CRC diagnosis are based on stool testing. Endogenous miRNAs encapsulated in exosomes are protected against RNases, in contrast to mRNAs or proteins that are rapidly degraded. For this reason, the detection of miRNA in stool is relatively easier. However, in order to ensure sensitivity and replicability, appropriate protocols, including material preparation, extraction, and quantitative analysis of miRNA, are required in this case [14]. Stool tests allow for earlier detection of tumor cells and most tumor markers, as compared to peripheral blood tests. Therefore, stool miRNA assays may be useful in detecting precancerous lesions [15]. Stool miRNA purification kits are commercially available, making it possible to obtain high-quality and high-purity nucleic acids for further analysis. However, methods that use stool miRNA molecules as biomarkers of CRC are still in their infancy. Although many recent studies indicate that stool miRNA tests have higher specificity, higher sensitivity and higher reproducibility than peripheral blood assays, no particular stool test has yet passed the preclinical phase. Therefore, it is necessary to carry out further studies and validation of methods based on miRNA derived from this material.

## 3. MiRNAs as Prognostic Biomarkers and Therapeutic Agents

MiRNA molecules also appear to be promising prognostic biomarkers, as has been shown so far in many preclinical and clinical studies [16,17,18,19,20,21,22]. The profiling of miRNA expression for prognostic purposes has been demonstrated in many human tumors, including: colorectal, pancreatic, ovarian, breast cancer and glioblastoma [15,23,24,25]. Since one type of miRNA molecule can influence the regulation of expression of many different genes, the use of these molecules in anti-cancer therapy also seems promising. Currently, there are two potential strategies: (1) the inhibition of oncogenic miRNAs and (2) the activation of suppressive miRNAs. Both strategies can be effective, as shown in preclinical studies. Direct inhibition involves antisense oligonucleotides used to sequester a given miRNAs. Modified antisense oligonucleotides used to inhibit miRNA in vivo are often referred to in the literature as antagomirs [26]. For example, a study conducted by Lanford et al. [27] published in Science in 2010 showed that the use of anti-miR-122 in chimpanzees chronically infected with hepatitis C virus (HCV) contributes to an improvement in liver disease. Currently, anti-miR-122 is in phase II clinical trials of HCV therapy in humans, and this miRNA-based therapy may possibly be included in clinical treatments in the coming years. The use of miRNA antagonists seems to be a promising form of therapy, as evidenced by the successful treatment of patients with chronic HCV infection [28]. Additionally, miRNAs can also be inhibited indirectly using a variety of chemical compounds [29]. Moreover, there are also studies on genetic knockout of miRNAs in cancer cells. These studies can provide valuable information about the role of miRNAs in cancer and contribute to the development of novel anti-cancer strategies. For example, Shi et al. [30] showed in mouse model that knockout of oncogenic *miR-21* causes an attenuated proliferation of colitis-associated CRC. In turn, Jiang and Hermeking [31] and Rokavec et al. [32] performed studies on suppressive miR-34 in mouse model. In these studies, the authors showed that genetic knockout of *miR-34a* and *miR-34b/c* can contribute to CRC progression.

## 4. MiR-21

One of the most intensively studied miRNA molecules is miR-21, which is often overexpressed in CRC [23,33]. MiR-21 lowers the expression of several different suppressor genes that influence various biological functions, such as proliferation, adhesion, angiogenesis, migration, metabolism, and apoptosis [34]. Therefore, aberrant miR-21 has potentially oncogenic properties. It is worth noting that some colorectal polyps transform into malignant tumors as a result of successive, consecutive genetic events. Many studies have shown that miR-21 is associated with the progression of polyps into malignant tumor, and that expression of this miRNA may be increased in CRC [35,36]. One study evaluated the expression of miR-21 in different stages of CRC in 39 surgically removed tumors and 34 polyps after endoscopic resection. Using in situ hybridization (ISH) of nucleic acids, expression of miR-21 was shown to be increased in non-malignant polyps in comparison with controls and was highest in advanced CRC tumors and also in adjacent stromal fibroblasts [36]. In another study, Bastaminejad et al. [37], using the qRT-PCR method, investigated the expression level of miR-21 in serum and stool samples from 40 patients with CRC and 40 healthy controls. The expression level of this miRNA was significantly up-regulated in serum (12.1-fold) and stool (10.0-fold) in CRC patients, compared to the control group. The sensitivity and specificity of serum miR-21 expression level were found to be 86.05% and 72.97%, respectively, and the sensitivity and specificity of stool miR-21 expression were 86.05% and 81.08%, respectively. The expression level of miR-21 was able to significantly distinguish CRC stages III–IV from stages I–II (according to the American Joint Committee on Cancer (AJCC) TNM staging system) in stool samples with a sensitivity and a specificity of 88.1% and 81.6%, respectively, and in serum samples with a sensitivity and a specificity of 88.10% and 73.68%, respectively. Significantly increased miR-21 expression was also demonstrated in stool samples of 88 CRC patients compared to control group of 101 healthy volunteers [38]. Similar results were obtained in 29 patients with CRC and eight healthy patients [39]. Therefore, the expression of this miRNA in tumor tissues as well as in serum and stool may be a potential and minimally invasive diagnostic biomarker of CRC.

MiR-21 overexpression is closely related to proliferation and lymph node metastases in CRC, which are important prognostic factors in this type of cancer. Analysis of the expression of miR-21 derived from CRC tissues may also be helpful in prognosis. Fukushima et al. [40] assessed the prognostic value of miR-21 in a group of 306 CRC patients. The authors found that high miR-21 expression was correlated with low overall survival (OS), as well as low disease-free survival (DFS) in CRC patients in Dukes stages B, C, and D [40]. In another study, the prognostic value of miR-21 was also considered in patients classified in the TNM staging system. After tumor tissues from 301 patients with varying degrees of CRC were investigated, a statistically significant correlation between miR-21 expression and prognosis was observed [23]. Moreover, high expression of this miRNA was associated with low OS. Oue et al. [23] demonstrated that miR-21 expression in tumor tissues is significantly increased in patients with tumors infiltrating adjacent organs (T4), compared to patients with tumors limited to the colon (T1–T3). The study also presented a similar relationship in patients with regional lymph node metastases present (N1) compared to patients without cancer in the lymph nodes (N0). Furthermore, high miR-21 expression in CRC patients was correlated with insensitivity to 5-fluorouracil (5-FU) treatment. Oue et al. [23] analyzed the expression of miR-21 in German (stage II, *n* = 145) and Japanese (stage I-IV, *n* = 156) cohorts of patients using the qRT-PCR method. MiR-21 overexpression was associated with poor prognosis in both Japanese (stage II/III) and German patients (stage II). These correlations were not dependent on other clinical data in a multivariate model. In addition, the use of adjuvant chemotherapy did not benefit patients with high miR-21 expression in both cohorts. Similar correlations were also obtained in other studies [16,19,41,42,43]. Moreover, Schetter et al. [16], using the ISH method, observed a high expression level of miR-21 in colonic epithelial cells in tumor tissues compared to adjacent non-tumor tissues. In turn, Nielsen et al. [42] detected miR-21 expression mainly in stromal fibroblasts adjacent to tumors. On the other hand, Xia et al. [44] showed in a meta-analysis of miR-21 expression profiles of 1174 CRC tissue samples that overexpression of this miRNA is associated with low OS, but there was no correlation with the carcinoembryonic antigen (CEA) level. Additionally, Chen et al. [45] and Peng et al. [46] in their meta-analysis studies showed that miR-21 expression in tumor tissues is associated with poor DFS and OS in CRC patients. However, Chen et al. [45] revealed the significant correlation between miR-21 expression and poor OS in studies based on the qRT-PCR analysis but not the fluorescence in situ hybrydization (FISH) method. Nonetheless, a few previous studies showed that a higher miR-21 expression level detected with the use of ISH method is associated with poor recurrence-free survival of CRC patients in stage II [41,47]. Moreover, in these studies, miR-21 expression was also detected mainly in stromal fibroblasts adjacent to tumors and only in a few samples in cancer cells. All studies discussed above indicated that the analysis of miR-21 expression in tumor tissues may be a potential, but certainly not an ideal biomarker of CRC prognosis.

The prognostic value of miR-21 expression in blood and stool of CRC patients is also the subject of intensive research. Kanaan et al. [48] observed significantly increased plasma levels of miR-21 in CRC patients. In turn, Toiyama et al. [33] evaluated the expression of miR-21 in a cell culture medium from two different CRC lines, in serum collected from 12 CRC patients and 12 healthy volunteers separately, and confirmed that this miRNA belongs to the secretory group of the miRNAs. The same research group subsequently measured miR-21 expression in 246 blood samples from CRC patients, 53 healthy volunteers, and 43 colorectal polyps. They also compared the expression of miR-21 in serum and tumor tissues in 166 paired samples. Statistically significant increase in serum miR-21 expression was observed in patients with benign polyps and in those with CRC. Moreover, a decrease in the expression of this miRNA in serum was observed in patients after surgical removal of the tumor [33]. Furthermore, many studies showed that expression of miR-21 both in tissue and serum samples of CRC patients is associated with lower OS and DFS [33,49]. However, Chen et al. [45] showed no significant association of serum miR-21 expression with poor OS of CRC patients in their meta-analysis studies (421 patients). In another study, it was shown that the increase in miR-21 expression in stool of CRC patients may be correlated with TNM classification [50]. Similarly, Bastaminejad et al. [37] revealed that increased expression of miR-21 was associated with AJCC TNM staging, more related with III and IV compared to I and II stages, in both serum and stool samples of 40 CRC patients. The above-mentioned studies show that miR-21 expression is associated with tumor size, metastases and low patient survival. Therefore, the expression of this miRNA in tumor tissues as well as in serum and stool may be a potential and minimally invasive prognostic biomarker of CRC.

## 5. MiR-29 Family

Another promising potential CRC biomarker is the miR-29 family, which includes three related miRNAs: miR-29a, miR-29b, and miR-29c. This family is associated with various molecular functions, such as the regulation of cell proliferation, cell senescence and tumor metastasis. Therefore, these molecules can participate in carcinogenesis and progression. It has been shown that the expression of miRNAs belonging to this family is altered in many different cancers [51]. Wang et al. [52] performed a study in 114 patients with CRC—56 subjects without metastasis and 58 with liver metastasis, which are commonly found in this type of cancer. The authors demonstrated that the expression of miR-29a in serum of patients with liver metastasis was significantly increased. In addition, a significantly increased expression of miR-29a was also observed in patients in stage T4, compared to T2. The authors concluded that miR-29a enables the early detection of liver metastasis in CRC [52]. In addition to early metastasis detection, miR-29a was also tested for potential use as a diagnostic biomarker for CRC. Huang et al. [53], using qRT-PCR, studied the expression of 12 miRNAs (miR-17-3p, miR-25, miR-29a, miR-92a, miR-134, miR-146a, miR-181d, miR-191, miR-221, miR-222, miR-223, and miR-320a) in the plasma of patients with advanced stages of colorectal neoplasia (CRC and advanced adenomas), compared to a group of healthy volunteers. It was shown that the expression of two miRNAs, miR-29a and miR-92a, can have a significant diagnostic value in CRC. For miR-29a, the area under the curve (AUC) was 0.844, while for miR-92a, the AUC was 0.838 in differentiating patients with CRC from healthy volunteers. The utility of both miRNAs was also demonstrated in differentiation of advanced adenomas and normal tissues. In this case, the AUC value for miR-29a was 0.769, while for miR-92a, it was 0.749. Overall, a receiver operating characteristic (ROC) analysis for both miRNAs showed an AUC of 0.883 (sensitivity = 83.0% and specificity = 84.7%) in differentiation of CRC and an AUC of 0.773 (sensitivity = 73.0% and specificity = 79.7%) in differentiation of advanced adenomas. Similarly, Al-Sheikh et al. [54] revealed up-regulation of miR-29 and miR-92, and down-regulation of miR-145 and miR-195 in 20 CRC patients (both in tissue and plasma) compared to controls. The above-mentioned results suggest that the determination of miR-29a and miR-92a expression in plasma may be a novel and potential biomarker in the diagnosis of CRC.

MiR-29b is also the member of miR-29 family. This miRNA inhibits proliferation and induces apoptosis in CRC cells. MiR-29b mediates the inhibition of the epithelial–mesenchymal transition. In many tumors that originate from epithelium, including CRC, the epithelial–mesenchymal transition is considered to be a key processes in initiation of metastasis. Li et al. [55] showed decreased expression of miR-29b in tissue and plasma samples obtained from CRC patients compared to controls. In addition, Basati et al. [56] showed down-regulation of miR-29b and miR-194 in serum samples obtained from 55 CRC patients compared to controls. Moreover, these authors showed a negative correlation between these miRNA expression and TNM stages.

MiR-29 is also a potential prognostic biomarker of CRC. Tang et al. [57] analyzed the expression of miR-29a and *KLF4* mRNA in 85 tumor tissues of CRC patients and CRC cell lines using the qRT-PCR method. It was shown that reduced expression of *KLF4* mRNA is associated with the presence of metastasis. Moreover, increased miR-29a expression indicated presence of metastasis and worsened prognosis of patients with CRC. It is known that *KLF4* is a target of miR-29a and that it acts to inhibit metastasis by reducing *MMP-2* and increasing E-cadherin expression [57]. The study mentioned above showed that high expression of miR-29a is associated with metastasis and poor prognosis. The predictive value of miR-29a was also shown in stage II CRC. Weissmann-Brenner et al. [58] performed studies on 110 CRC patients (51 with stage I cancer and 59 patients with stage II cancer according to the AJCC TNM staging system) using miRNA microarrays and verified the microarray results using the qRT-PCR technique afterwards. RNA was extracted from formalin-fixed paraffin-embedded tumor tissues. The authors defined a poor prognosis as a recurrence of the disease within 36 months of surgery. Patients with a good prognosis (*n* = 87; 79%) and a poor prognosis (*n* = 23; 21%) were compared. There were no statistically significant differentially expressed miRNAs between good- and poor-prognosis stage I CRC patients, among the set of 903 analyzed miRNAs. On the other hand, the expression of miR-29a was significantly higher in stage II CRC patients with good prognosis compared to the poor-prognosis group. High expression of this miRNA was associated with longer DFS in both univariate and multivariate analysis. In case of miR-29a expression, a positive predictive value (PPV) for non-recurrence of the disease was 94% (two cases out of 31). Differences in the miR-29a expression were confirmed using qRT-PCR, and this method showed the effect of overexpression of this miRNA on prolonging of the DFS. This study demonstrated a significant association of the miR-29a expression level with the risk of CRC recurrence in stage II patients. For the patients in stage I, no such correlation was demonstrated. Moreover, significantly decreased expressions of miR-29a and miR-29c were reported in tumor tissues in 43 early-recurrence patients compared to the control group [59]. Although increased expressions of both miR-29a and miR-29c were associated with better outcome after 12 months of therapy, the authors suggested that only miR-29a may be used as a predictor marker for an early recurrence of the disease. The low PPV of miR-29c in this case may result from short follow-up time of the patients and the small study group [59]. In another study on 245 patients by Inoue et al. [60], the expression of miR-29b level in tumor tissues was used to divide the patients into two groups. The reference value was the median expression of this miRNA. The authors concluded that higher expression of miR-29b is associated with higher five-year DFS and OS values. Analysis of the severity of the disease showed that the miR-29b expression reflects the five-year DFS and has a significant prognostic value, but only in patients with stage III CRC. In addition, the low level of miR-29b expression was also a predictor of lymph node metastasis. These findings confirmed the prognostic value of this miRNA in stage III CRC patients. In another study, Yuan et al. [61] studied the expression of miR-29b in tumor tissue and adjacent normal mucosa samples of 41 patients using the qRT-PCR method. The authors found a significant decrease in the expression of this miRNA in CRC and concluded that the level of miR-29b may be associated with the size of a tumor, clinical status and lymph node metastasis. Basati et al. [56] studied 55 serum samples of CRC patients and revealed that lower expression of miR-29b is correlated with poor prognosis. In turn, Ulivi et al. [62] showed that higher miR-29b level in plasma samples is associated with longer progression-free survival (PFS) and OS of metastatic CRC patients treated with bevacizumab-based chemotherapy. As indicated by numerous studies, analysis of changes of miR-29 expression may be helpful in assessing an early recurrence and evaluating DFS in CRC patients.

## 6. MiR-34 Family

There are also studies on miR-34, a group of miRNAs that includes miR-34a, miR-34b and miR-34c. These molecules show suppressor properties and are regulated by p53 protein and DNA hypermethylation. The miR-34 group influences various processes that occur in tumor cells such as differentiation, drug resistance and metastasis [63]. For example, miR-34a overexpression inhibits NOTCH signaling and suppresses symmetric cell division, which prevents expansion of the CRC stem cell niche [64]. Wu et al. [65] studied the possibility of using miR-34 as a potential diagnostic biomarker for CRC. The authors showed abnormal methylation of miR-34a, miR-34b, and miR-34c genes in tissue and stool samples of 82 CRC patients. In turn, Aherne et al. [66] reported higher expression of miR-34a in plasma samples obtained from CRC patients compared to controls.

MiR-34 expression has also been found to be useful for CRC prognosis. The usefulness of miR-34 as a biomarker of a recurrence of the disease in two independent groups of 268 CRC patients was evaluated. It was shown that the miR-34a expression in CRC tissues is directly proportional to DFS, and therefore, this molecule may be a good prognostic factor in assessing the risk of the recurrence of CRC. In addition, the expression of miR-34a was significantly higher in patients with high expression of p53 compared to those with low expression of this protein. The authors suggested that miR-34 inhibits the growth and invasiveness of CRC in p53-dependent manner, which allows this miRNA to be used as a potential biomarker for a recurrence in patients with stage II and stage III CRC [67]. The PAR2 receptor also plays an important role in the progression of CRC. Previous reports have indicated that miR-34a expression is inhibited by PAR2, which results in increased synthesis of cyclin D1 and transforming growth factor β (*TGF-β*) in CRC cells [68]. Furthermore, silencing of miR-34a expression through promoter methylation in CRC tissues is associated with the occurrence of metastases [69]. In other studies, a lower expression of this miRNA was observed in some patients with CRC in tissue [70] and serum/plasma samples [71], which suggests that miR-34a may play a role in the progression of this cancer. Li et al. [72] showed that lower expression of miR-34a in CRC tissues is correlated with the lymph node metastasis and TNM stage. Zhang et al. [73] performed studies on 103 CRC tissue samples and showed that miR-34a expression was down-regulated compared to adjacent normal mucosa samples with the use of ISH technique. Moreover, these authors indicated that the lower expression of this molecule is correlated with more distant metastasis and shorter OS time. Studies on the effect of the miR-34 group on the prognosis of CRC have also been performed. The purpose of these studies was to determine the relationship between the miR-34b and miR-34c expression in CRC tissues and the development of this disease. Samples were obtained from 159 American and 113 Chinese patients with stages I–IV CRC. Using the qRT-PCR method, Hiyoshi et al. [74] showed an increased expression of miR-34b and miR-34c in advanced CRC, which was associated with poor prognosis in both study groups. Similarly to miR-34a, the expressions of miR-34b and miR-34c are also regulated by p53 protein at the transcriptional level. The results of all of these studies show that miR-34 may be an interesting prognostic tool and may be used to assess the risk of a recurrence in patients with CRC.

Furthermore, recent studies have determined the possibility of using miR-34 as a potential therapeutic agent. A low level of this miRNA was observed in DLD-1 CRC cell line that was resistant to 5-FU. Restoration of the miR-34a expression caused the sensitization of cells to 5-FU treatment and resulted in an inhibition of cell growth [75]. Moreover, Sun et al. [76] showed that miR-34a expression was down-regulated in blood samples of CRC patients after oxaliplatin-based chemotherapy. The negative correlation between miR-34a and *TGF-β/SMAD4* expression is also noted. The authors suggested that miR-34a and *TGF-β/SMAD4* expression changes can lead to activation of macroautophagy and oxaliplatin resistance in CRC cells.

## 7. MiR-124

MiR-124 is known to inhibit cell proliferation, metastasis and invasion in CRC. This miRNA not only down-regulates rho-associated protein kinase 1 (ROCK1) [77], which functions as an oncogene, but also inhibits the activity of cyclin-dependent kinase 4 (CDK4), which is responsible for cell cycle progression at the G1/S checkpoint [78]. Studies have shown that, in CRC cells, the expression of miR-124 is silenced through DNA methylation [79,80]. Since *miR-124* gene is more likely to be methylated in CRCs compared to other tumors, DNA methylation status of this miRNA may be used as a specific marker for CRC. Harada et al. [79] performed the detection of DNA methylation in bowel lavage fluid for CRC screening. These authors analyzed DNA methylation status in a total of 508 patients—56 with CRC, 53 with advanced adenoma, 209 with minor polyp, and 190 healthy individuals. Three genes showed the greatest sensitivity for CRC detection (*miR-124-3*, *LOC386758*, and *SFRP1*) after training set analysis (*n* = 345). A scoring system based on the methylation of these three genes achieved 82% sensitivity and 79% specificity, and the AUC was 0.834. These results were subsequently validated in an independent test set (*n* = 153; AUC = 0.808). In another study, Xi et al. [77] investigated the expression of ROCK1 and miR-124 in CRC patients using 68 paired tissue specimens (38 cases of non-metastatic CRC and 30 cases of metastatic CRC). The use of qRT-PCR revealed that expression of miR-124 was higher in normal compared with CRC tissues, and in non-metastatic compared to metastatic CRC tissues. In contrast, ROCK1 was significantly overexpressed in CRC compared with control tissues and between metastatic tissues and non-metastatic CRC tissues. The above-mentioned results suggest that miR-124, as a tumor suppressor, may play a role in tumor growth and metastasis. Recently, the miR-124a level has also been studied in 40 patients with ulcerative colitis (without colorectal cancer), four patients with CRC or inflammatory dysplasia, eight patients with CRC (without inflammatory background), and 12 healthy volunteers. It was found that miR-124a-1, miR-124a-2, and miR-124a-3 genes are methylated in tumor tissues. The authors suggested that the methylation of miR-124a-3 occurring during oncogenesis in patients with ulcerative colitis can be used to evaluate the individual’s risk of developing cancer [81].

MiR-124 may also be a prognostic marker for CRC. Wang et al. [82] studied 96 tumor tissue samples and showed that down-regulated expression of miR-124 is correlated with poor OS and DFS in CRC patients. Moreover, Jinushi et al. [83] showed that higher expression of miR-124-5p (both in plasma and tissues) was associated with better prognosis of CRC patients. In turn, Slattery et al. [84] performed studies in 1893 patients with CRC. The authors found that miR-124-3p belongs to infrequently highly expressed miRNAs in tumor tissues and showed that up-regulation of miR-124-3p can worsen prognosis of these CRC patients.

## 8. MiR-130b

The direct target of miR-130b is the PPARγ receptor, of which inhibition results in the regulation of the expression of cadherin E, vascular endothelial growth factor (VEGF) and phosphatase and tensin homolog (PTEN). Since tissue miR-130b overexpression was observed in stage III and IV CRCs, it is suggested that miR-130b-PPARγ signaling may play a significant role in increasing tumor malignancy. Furthermore, evaluating the expression of proteins in this pathway, including miR-130b, may be a promising prognostic biomarker in CRC [85]. Finally, a study performed in 53 cancer and non-cancerous samples [86] suggested miR-130a as a good biomarker of CRC because of correlation with TNM staging and lymph node metastasis.

## 9. MiR-155

The sequence of this miRNA is located in a non-coding region of MIR155 host gene (*BIC*). Altered expression of miR-155 has been observed in many different tumors, and is associated with severity of disease, progression, and response to treatment. Interestingly, Sempere et al. [87] using the ISH method observed that miR-155 expression is detected mainly in tumor-infiltrating immune cells. Lv et al. [88] examined the possibility of using serum miR-155 expression as a diagnostic tool. Using qRT-PCR, they measured the expression levels of miR-155 in 146 CRC patients and 60 healthy controls. Serum miR-155 was up-regulated in CRC patients compared with the matched healthy controls. Moreover, ROC curve analysis indicated that miR-155 is a suitable marker for discriminating CRC patients from healthy controls, with an AUC of 0.776. Therefore, this molecule can be used as a potential tumor biomarker in the diagnosis of CRC.

MiR-155 can also play an important role in CRC prognosis. Shibuya et al. [43] showed that patients with an increased expression of miR-155 in tumor tissues were characterized by shorter OS and DFS, compared to those with lower expression of this miRNA. In another study, multivariate analysis also demonstrated a relationship between the level of miR-155 expression and poor prognosis in CRC, depending on the severity of the disease. The control group consisted of 60 healthy volunteers, while the experimental group consisted of 146 patients. The authors did not observe changes in the miR-155 serum level in patients with stage I CRC, but reported a statistically significant overexpression of this miRNA in patients with stages II–IV of the disease [88]. In another study, the serum CEA level and miR-155 expression were measured in tissues that were obtained before and after surgery of 84 CRC patients. It is well known that CEA is used for determining the prognosis, evaluating the effectiveness of therapy and monitoring the recurrence of CRC. In this study, the miR-155 expression was observed to be significantly increased in patients with CRC. This is associated with metastases and a recurrence of the disease [89]. Therefore, the evaluation of miR-155 expression in association with serum CEA may provide additional diagnostic information and enable a more accurate assessment of the risk of the metastasis of CRC. A statistically significant increase in miR-155 expression in tumor tissues, compared to normal samples, was also observed by Zhang et al. [90] in a study of 76 patients with CRC. In addition, the authors observed a correlation between miR-155 expression and lymph node and distant metastases and disease progression. Moreover, they observed that miR-155 overexpression inhibited E-cadherin expression and positively regulated zinc finger E-box binding homeobox 1 protein (ZEB-1), which resulted in an increased cell migration and metastases. Similarly, Qu et al. [91] revealed association of miR-155-5p expression in tumor tissues with location, grade of tumor, TNM stage and distant metastasis. Ulivi et al. [62] in multivariate analysis also showed that increased expression of circulating miR-155 is associated with shorter PFS and OS in metastatic CRC patients treated with bevacizumab-based chemotherapy. The above-mentioned observations indicate that miR-155 may play a role in the development and metastasis of CRC.

## 10. MiR-224

Recent reports have revealed that miR-224 may influence many processes that are associated with tumor cell growth and development, such as proliferation, growth, differentiation and apoptosis [92]. Some groups have investigated the expression of miR-224 in CRC patients. Zhu et al. [93] found significantly lower miR-224 levels in feces from CRC patients than these from normal volunteers in their retrospective analysis of miR-224 levels in fecal samples from 80 CRC patients and 51 normal controls. The authors suggested that the miR-224 expression level in feces can be used for screening and early diagnosis of CRC.

MiR-224 is also a potential prognostic biomarker in CRC. Zhang et al. [94] evaluated the clinical and pathological information of 40 patients with a recurrence and 68 patients without a recurrence within three years after a surgical intervention. Moreover, using the qRT-PCR and Western blot methods, the authors analyzed samples from all 108 patients with stages I and II CRC. They showed that miR-224 is involved in the regulation of *SMAD4* protein, which is involved in cell signaling. *SMAD4*, together with other proteins from this family, forms a DNA-binding complex that acts as a transcription factor. Furthermore, a significant increase in miR-224 expression in CRC tissues was observed in the study and this change was associated with a higher risk of a recurrence and a shorter DFS. In another study, Ling et al. [95] showed that miR-224 is an activator of metastasis and that the target of this miRNA is *SMAD4*. The authors concluded that an evaluation of miR-224 alone or an evaluation of miR-224 together with *SMAD4* may be an independent prognostic marker in patients with CRC. MiR-224 expression in tumor tissues and its association with the survival of patients were evaluated in 449 CRC patients. In this study, the patients were divided into two groups. These two groups were characterized by low and high levels of miR-224 expression, respectively. A shorter OS and survival time without metastases were observed in patients with miR-224 overexpression. Moreover, Wang et al. [96] showed an inverse correlation between *SMAD4* and miR-224 expression in tumor tissues of 40 CRC patients. These authors also revealed that miR-224 can regulate USP3 expression and its higher expression is associated with poor prognosis. In turn, Liao et al. [97] observed a statistically significant increase in the expression of this miRNA in tumor tissues of patients with an aggressive CRC phenotype and poor prognosis. In another study, miR-224 expression was evaluated in 79 patients with CRC and 18 healthy volunteers. The authors observed a significant inhibition of miR-224 expression in tumor tissues. Since the molecular target of miR-224 is *CDC42*, a lower expression of this miRNA reduces tumor cells migration. In general, the study indicated an important role of miR-224 in inhibiting migration of CRC cells. The authors concluded that miR-224 may be a promising biomarker in evaluating development of CRC [98]. Furthermore, Zhang et al. [99] showed in their meta-analysis that the increased level of miR-224-5p is correlated with poor OS of CRC patients.

## 11. MiR-378

MiR-378 is known to play a significant role in development of different types of cancer, including CRC. Current studies have shown that miR-378 is overexpressed in CRC cells and its targets include *FUS-1* and *SUFU* suppressor genes [100,101]. In addition, miR-378 is involved in tumor progression by promoting cell survival, migration and angiogenesis [102]. Significant differences in the level of blood and tumor miR-378 expression between oncological and healthy subjects were observed. Zanutto et al. [103] analyzed miRNA expression in serum samples from 65 CRC patients and 70 healthy volunteers, and found significantly increased miR-378 levels in CRC patients compared to the control group. At the same time, the authors observed a statistically significant decrease in the expression of this miRNA after the surgical removal of the tumor. Similar results were obtained in patients who had no recurrence of the disease within four to six months after surgery. The results suggest that serum miR-378 levels may be useful not only for differentiating CRC patients from healthy subjects, but that miR-378 is synthesized in the tumor tissue and its concentration is associated with tumor mass and possible recurrence. In turn, Wang et al. [104] qualified miR-378 as a tumor suppressor after analyzing miRNA expression in 47 CRC samples that were matched with normal tissue samples. In another study, Zhang et al. [105] observed an inhibition of miR-378 expression in 84 CRC samples compared to normal mucosal samples. Similarly, Zeng et al. [106] showed lower miR-378 expression in 27 CRC samples compared to paired adjacent normal samples. There have also been studies that showed an association of a reduced expression of miR-378 in cancer tissues with increased tumor size, metastasis and shorter OS in patients with CRC [105,107]. The above-mentioned results suggest that miR-378 may play an important role in carcinogenesis and may have clinical value as a potential biomarker of CRC.

## 12. Other miRNAs

Many recent studies attempted to identify other miRNAs in tumor tissues or plasma/serum samples as the potential diagnostic, prognostic or predictive biomarkers of CRC, e.g., miR-17, miR-19a, miR-20a [108], miR-22 [109], miR-24-3p [110], miR-26a, miR-26b [111], miR-9 [112,113,114], miR-106a [115], miR-122, miR-200 [116], miR-125a-3p [117], miR-126-3p [118], miR-139-3p [119], miR-139-5p [120], miR-148a, miR-625-3p [121], miR-181a, miR-181b [122], miR-181c [123], miR-181d [124], miR-193a-3p [125], miR-200c [126,127], miR-196b-5p [126], miR-223 [108,113], miR-375, miR-760 [112], miR-506, miR-4316 [128], miR-1290 [129]. In addition, Kiss et al. [130] using microarray and qRT-PCR techniques revealed that miR-92b-3p, miR-3156-5p, miR-10a-5p, miR-125a-5p may be used as predictive biomarkers of response to bevacizumab/FOLFOX therapy of CRC patients with metastasis. Similarly, Fiala et al. [118] showed that miR-126-3p expression is correlated with response to bevacizumab/FOLFOX treatment of CRC patients with metastasis. On the other hand, some studies showed only altered expression of various miRNAs in CRC tissues/serum samples compared to controls [131,132]. The significance of miRNAs as diagnostic and prognostic biomarkers in CRC is summarized in Table 2.

## 13. MiRNA Panels

More researchers are focusing on finding miRNA sets that may be used as potential diagnostic or prognostic markers due to the fact that the expression of a single miRNA may not have sufficient specificity and sensitivity to distinguish CRC stages or CRC patients from healthy controls. Recently, interesting study was performed by Pan et al. [133], in which the expression level of 30 miRNAs in plasma samples was analyzed with the use of qRT-PCR. These authors showed that analysis of plasma expression level of five miRNAs, such as miR-15b, miR-17, miR-21, miR-26b, and miR-145, together with CEA, can improve diagnostic accuracy of CRC (AUC = 0.85 in the training cohort, AUC = 0.818 in the validation cohort). In turn, Guo et al. [134] selected a 5-miRNA set (miR-1246, miR-202-3p, miR-21-3p, miR-1229-3p, and miR-532-3p) from 528 miRNAs in serum and revealed that these miRNA panels have good sensitivity and specificity to distinguish CRC patients from colorectal adenoma patients (AUC = 0.951, sensitivity = 94.4%, specificity = 84.7%) and healthy controls (AUC = 0.960, sensitivity = 91.6%, specificity = 91.7%). Zhu et al. [135] showed that a 3-serum miRNA set (miR-19a-3p, miR-21-5p, and miR-425-5p) can be useful in diagnosis of CRC (AUC = 0.886 in the training phase, AUC = 0.768 in the validation phase, AUC = 0.783 in the combined training and validation phases, and AUC = 0.830 in the external validation phase). Wang et al. [136] also evaluated a 3-plasma miRNA set (miR-409-3p, miR-7, and miR-93) with diagnostic potential for CRC patients in Dukes stages A–D (AUC = 0.866 in the training phase, sensitivity = 91%, specificity = 88%; AUC = 0.897 in the validation phase, sensitivity = 82%, specificity = 89%) and also for non-metastatic CRC patients in Dukes stages A and B (AUC = 0.809 in the training phase, sensitivity = 85%, specificity = 88%; AUC = 0.892 in the validation phase, sensitivity = 82%, specificity = 89%), and for metastatic CRC patients in Dukes stages C and D (AUC = 0.917 in the training phase, sensitivity = 96%, specificity = 88%; AUC = 0.865 in the validation phase, sensitivity = 91%, specificity = 88%). Similar results were obtained in CRC patients when compared to age-matched healthy controls (for CRC patients in Dukes stages A–D: AUC = 0.894, sensitivity = 90%, specificity = 96%; in Dukes stages A and B: AUC = 0.850, sensitivity = 85%, specificity = 96%; and in Dukes stages C and D: AUC = 0.937, sensitivity = 95%, specificity = 96%). In turn, Kanaan et al. [137] observed that a 3-plasma miRNA set (miR-431, miR-15b, and miR-139-3p) can distinguish stage IV CRC from controls with high specificity and sensitivity (AUC = 0.896, sensitivity = 93%, specificity = 74%). They also found that a 5-miRNA set (miR-331, miR-15b, miR-21, miR-142-3p, and miR-339-3p) may be used to distinguish colorectal adenoma patients from CRC patients (AUC = 0.856, sensitivity = 91%, specificity = 69%), and CRC patients may be distinguished from healthy controls with the use of a 2-miRNA set (miR-431, and miR-139-3p) (AUC = 0.829, sensitivity = 91%, specificity = 57%). Wikberg et al. [138] used a 4-plasma miRNA set (miR-18a, miR-21, miR-22, and miR-25) to diagnose CRC (in all CRC stages, AUC = 0.93, sensitivity = 81% and 67% at 80% and 90% specificity, respectively; in CRC stages I–II, AUC = 0.92, sensitivity = 88% and 73% at 80% and 90% specificity, respectively; and in CRC stages, III–IV AUC = 0.85, sensitivity = 68% and 57% at 80% and 90% specificity, respectively). It is worth noticing that miR-21 is present in majority of miRNA sets discussed above. Additionally, Wikberg et al. [138] showed that miR-21 expression was increasing during three years before CRC diagnosis. Interestingly, Yang et al. [139] revealed that a 6-miRNA set (miR-7, miR-93, miR-195, miR-141, miR-494, and let-7b) in tumor tissues together with six clinicopathological factors (the Union for International Cancer Control (UICC) stage, location, type of tumor, vascular invasion, perineural invasion, and lymph node metastasis) can be used as potential prognostic markers of CRC recurrence within 12 months after surgery (AUC = 0.948, sensitivity = 89.4%, specificity = 88.9%). Moreover, based on literature data, Liu et al. [140] found 63 miRNAs, which can have diagnostic value for CRC. Then, using qRT-PCR technique, the authors analyzed the expression of five miRNAs: miR-21, miR-29a, miR-92a, miR-125b and miR-223 in serum samples obtained from 85 CRC patients and revealed that expression levels of these miRNAs were up-regulated in CRC samples compared to healthy controls. However, there were no differences in expression levels between TNM stages. The authors suggested that analysis of these five miRNAs together has higher diagnostic value than expression analysis of a single miRNA (AUC = 0.952, sensitivity = 84.7%, specificity = 98.7%). Interestingly, the authors also compared diagnostic value of CEA and CA19-9 with gas chromatography-mass spectrometry metabolomic data. The results discussed above show that miRNA panels in general have better sensitivity and similar specificity when compared to CRC screening tests currently used in clinical practice, such as FOBT (sensitivity = 64.3% (range: 35.6–86%), specificity = 90.1% (range: 89.3–90.8%)) and fecal immunochemical tests (FITs) (sensitivity = 81.8% (range: 47.8–96.8%), specificity = 96.9% (range: 96.4–97.4%)) [141,142]. The significance of miRNA panels as diagnostic and prognostic biomarkers in CRC is summarized in Table 3.

## 14. Conclusions

Our paper presents the latest reports on the diagnostic and prognostic values of selected miRNAs in CRC. Although the number of published papers that describe miRNAs as potential biomarkers for CRC has increased significantly over the past decade, clinical knowledge still remains fragmented. Only two miRNAs (miR-21 and miR-29) have been described in more detail in many previous studies. However, it is necessary to conduct further prospective validation studies before translating the knowledge into clinical use. Most of the current findings are from preliminary studies, which are often not free of methodological limitations such as small sample size, lack of detailed patient information, untested replicability, and statistical errors. It is also worth noticing that using the expression of a single miRNA as a diagnostic or prognostic biomarker of CRC is often limited due to insufficient specificity and sensitivity. Currently, many groups of researchers are investigating miRNA panels as CRC biomarkers, which appears to be a more promising strategy than the use of single miRNA tests. The development of panels containing many miRNA biomarkers seems to be essential and may enable more accurate diagnoses and prognoses of CRC in the future. However, the cost–benefit issue is also important in this case. In addition, for every potential miRNA biomarker, it is necessary to understand its molecular and biological functions as well as the mechanisms that are associated with its regulation. Understanding these processes is key to clinical application and identification of new therapeutic targets.

## Figures and Tables

**Table 1:** Possibilities and advantages of using miRNA for diagnostic, therapeutic and prognostic purposes in cancer diseases.

**Possible Applications of miRNA:**
Predictors of response to therapy and prognostic biomarkers
Use of miRNA as a drug—modulation of gene expression
Prediction and detection of metastases or non-invasive tumor phenotypes
Cancer diagnostics—detection of tumor specific miRNA signatures
**Advantages of Using miRNA:**
Easy detection in various biological materials (serum/plasma, cerebrospinal fluid, faeces)
High stability of miRNA molecules
Ability to determine specific types of cancer, and predict response to therapy and prognosis based on miRNA expression profile
Potential for use as antagonists in cancer therapy

**Table 2:** The significance of miRNAs as diagnostic and prognostic biomarkers of colorectal cancer.

MiRNA	Biomarker Type	Regulation in CRC	Source of miRNA	Cohort Size	Correlation/Differentiation	Detection Method	Authors
miR-17	diagnostic	up-regulation	serum	*n* = 190	control vs. CRC	qRT-PCR	Zekri et al. [108]
miR-17-5p	diagnostic	up-regulation	serum	*n* = 39	control vs. CRC	qRT-PCR	Fu et al. [114]
miR-19a	diagnostic	up-regulation	serum	*n* = 190	control vs. CRC	qRT-PCR	Zekri et al. [108]
miR-20a	diagnostic	up-regulation	serum	*n* = 190	control vs. CRC	qRT-PCR	Zekri et al. [108]
miR-21	diagnostic	up-regulation	plasma, tissues	*n* = 116	control vs. CRC	microfluidic array technology, qRT-PCR	Kanaan et al. [48]
miR-21	diagnostic	up-regulation	tissues	*n* = 73	control vs. CRC vs. polyps	ISH	Yamamichi et al. [36]
miR-21	diagnostic	up-regulation	serum, stool	*n* = 80	TNM stages	qRT-PCR	Bastaminejad et al. [37]
miR-21	diagnostic	up-regulation	stool, tissues	*n* = 246	control vs. CRC vs. polyps	qRT-PCR	Wu et al. [38]
miR-21	diagnostic	up-regulation	stool	*n* = 55	TNM stages	miRNA microarrays, qRT-PCR	Ahmed et al. [50]
miR-21	diagnostic	up-regulation	stool, tissues	*n* = 37	control vs. CRC vs. adenomas	qRT-PCR, miRNA microarrays	Link et al. [39]
miR-21	diagnostic, prognostic	up-regulation	serum, tissues	*n* = 279	TNM stages, tumor size, distant metastasis, poor survival	qRT-PCR	Toiyama et al. [33]
miR-21	prognostic	up-regulation	tissues	*n* = 301	poor survival	qRT-PCR	Oue et al. [23]
miR-21	prognostic	up-regulation	tissues	*n* = 306	liver metastasis, Dukes’ stage, shorter OS, shorter DFS	qRT-PCR	Fukushima et al. [40]
miR-21	prognostic	up-regulation	tissues	*n* = 197	poor survival, poor therapeutic outcome	miRNA microarrays, qRT-PCR, ISH	Schetter et al. [16]
miR-21	prognostic	up-regulation	tissues	*n* = 46	shorter DFS	qRT-PCR	Kulda et al. [19]
miR-21	prognostic	up-regulation	tissues	*n* = 520	inferior recurrence-free cancer-specific survival	ISH	Kjaer-Frifeldt et al. [41]
miR-21	prognostic	up-regulation	tissues	*n* = 234	shorter DFS	ISH	Nielsen et al. [42]
miR-21	prognostic	up-regulation	tissues	*n* = 156	liver metastasis, shorter OS, shorter DFS	qRT-PCR	Shibuya et al. [43]
miR-21	prognostic	up-regulation	tissues	*n* = 277	shorter RFS	ISH	Kang et al. [47]
miR-22	prognostic	down-regulation	tissues	*n* = 193	shorter OS	qRT-PCR	Li et al. [109]
miR-24-3p	prognostic	up-regulation	tissues	*n* = 268	shorter OS, shorter DFS	qRT-PCR	Kerimis et al. [110]
miR-26a/b	prognostic	down-regulation	tissues	*n* = 58	shorter OS	qRT-PCR	Li et al. [111]
miR-29	diagnostic	up-regulation	tissues, plasma	*n* = 40	control vs. CRC	qRT-PCR	Al-Sheikh et al. [54]
miR-29a	diagnostic	up-regulation	plasma	*n* = 209	control vs. CRC	qRT-PCR	Huang et al. [53]
miR-29a	diagnostic	down-regulation	stool	*n* = 131	control vs. CRC	qRT-PCR	Zhu et al. [93]
miR-29a	diagnostic	up-regulation	serum	*n* = 74	liver metastasis	qRT-PCR	Wang and Gu [52]
miR-29a	prognostic	down-regulation	tissues	*n* = 110	poor prognosis	miRNA microarrays, qRT-PCR	Weissmann-Brenner et al. [58]
miR-29b	diagnostic	down-regulation	tissues, plasma	*n* = 600	control vs. CRC	qRT-PCR	Li et al. [55]
miR-29b	diagnostic, prognostic	down-regulation	serum	*n* = 110	TNM stages	qRT-PCR	Basati et al. [56]
miR-29b	prognostic	down-regulation	tissues	*n* = 245	shorter OS, shorter DFS	qRT-PCR	Inoue et al. [60]
miR-29b	prognostic	down-regulation	plasma	*n* = 52	shorter OS, shorter PFS	qRT-PCR	Ulivi et al. [62]
miR-34a	diagnostic	up-regulation	plasma	*n* = 185	polyps vs. advanced cancer	qRT-PCR	Aherne et al. [66]
miR-34a	diagnostic, prognostic	methylation	tissues, stool	*n* = 142	lymph metastasis, control vs. CRC	methylation-specific PCR	Wu et al. [65]
miR-34a	prognostic	down-regulation	tissues	*n* = 268	shorter DFS, recurrence	qRT-PCR	Gao et al. [67]
miR-34a	prognostic	methylation	tissues	*n* = 94	liver metastasis	methylation-specific PCR	Siemens et al. [69]
miR-34a	prognostic	down-regulation	tissues	*n* = 176	poor prognosis	qRT-PCR	Li et al. [72]
miR-34a	prognostic	down-regulation	tissues	*n* = 103	distant metastasis	qRT-PCR, ISH	Zhang et al. [73]
miR-34a/b/c	prognostic	up-regulation	tissues	*n* = 272	poor prognosis	qRT-PCR	Hiyoshi et al. [74]
miR-34b/c	diagnostic	methylation	tissues, stool	*n* = 142	control vs. CRC	methylation-specific PCR	Wu et al. [65]
miR-92a	diagnostic	up-regulation	tissues, stool, plasma	*n* = 907	control vs. CRC	qRT-PCR	Chang et al. [113]
miR-92a	diagnostic, prognostic	up-regulation	serum	*n* = 91	control vs. CRC, TNM stages, poor prognosis	qRT-PCR	Elshafei et al. [112]
miR-92a-3p	diagnostic	up-regulation	serum	*n* = 39	control vs. CRC	qRT-PCR	Fu et al. [114]
miR-106a	diagnostic	up-regulation	tissues, plasma	*n* = 84	control vs. CRC	qRT-PCR	He et al. [115]
miR-122	prognostic	up-regulation	plasma	*n* = 543	shorter RFS, shorter OS	qRT-PCR	Maierthaler et al. [116]
miR-124	prognostic	down-regulation	tissues	*n* = 96	shorter OS, shorter DFS	qRT-PCR	Wang et al. [82]
miR-124-3p	prognostic	up-regulation	tissues	*n* = 1893	increased likelihood of dying	miRNA microarrays	Slattery et al. [84]
miR-124-5p	prognostic	down-regulation	tissues	*n* = 71	shorter OS	qRT-PCR	Jinushi et al. [83]
miR-125a-3p	diagnostic	down-regulation	tissues	*n* = 35	control vs. CRC	qRT-PCR	Liang et al. [117]
miR-126-3p	prognostic	down-regulation	tissues	*n* = 63	shorter OS, shorter PFS	qRT-PCR	Fiala et al. [118]
miR-126-5p	prognostic	down-regulation	tissues	*n* = 63	shorter OS, shorter PFS	qRT-PCR	Fiala et al. [118]
miR-130b	prognostic	up-regulation	tissues	*n* = 80	poor prognosis	miRNA microarrays	Colangelo et al. [85]
miR-139-3p	diagnostic	down-regulation	tissues, serum	*n* = 249	control vs. CRC	qRT-PCR	Ng et al. [119]
miR-139-5p	prognostic	up-regulation	tissues, serum	*n* = 433	shorter RFS	miRNA microarrays, qRT-PCR	Miyoshi et al. [120]
miR-148a	prognostic	down-regulation	tissues	*n* = 54	shorter PFS	qRT-PCR	Baltruskeviciene et al. [121]
miR-150	diagnostic	down-regulation	plasma	*n* = 185	adenomas vs. advanced cancer	qRT-PCR	Aherne et al. [66]
miR-155	prognostic	up-regulation	serum	*n* = 206	shorter OS, shorter PFS	qRT-PCR	Lv et al. [88]
miR-155	prognostic	up-regulation	tissues	*n* = 156	shorter OS, shorter DFS	qRT-PCR	Shibuya et al. [43]
miR-155	prognostic	up-regulation	tissues	*n* = 84	poor prognosis	qRT-PCR	Hongliang et al. [89]
miR-155	prognostic	up-regulation	tissues	*n* = 76	lymph node and distant metastases	qRT-PCR	Zhang et al. [90]
miR-155-5p	prognostic	up-regulation	tissues	*n* = 372	metastasis	qRT-PCR	Qu et al. [91]
miR-155-5p	prognostic	down-regulation	plasma	*n* = 52	shorter OS, shorter PFS	qRT-PCR	Ulivi et al. [62]
miR-181c	prognostic	up-regulation	tissues	*n* = 147	shorter RFS	miRNA microarrays, qRT-PCR	Yamazaki et al. [123]
miR-181d	prognostic	up-regulation	tissues	*n* = 40	metastasis	qRT-PCR	Guo et al. [124]
miR-193a-3p	prognostic	down-regulation	tissues	*n* = 96	shorter OS	miRNA microarrays, qRT-PCR	Lin et al. [125]
miR-196-5p	prognostic	down-regulation	tissues	*n* = 48	shorter OS	miRNA microarrays, qRT-PCR	Li et al. [126]
miR-200	prognostic	down-regulation	plasma	*n* = 543	shorter RFS, shorter OS	qRT-PCR	Maierthaler et al. [116]
miR-200c	prognostic	down-regulation	tissues	*n* = 48	shorter OS	miRNA microarrays, qRT-PCR	Li et al. [126]
miR-200c	prognostic	up-regulation	serum	*n* = 90	shorter OS	qRT-PCR	Tayel et al. [127]
miR-223	diagnostic	down-regulation	stool	*n* = 131	control vs. CRC	qRT-PCR	Zhu et al. [93]
miR-223	diagnostic	up-regulation	serum	*n* = 190	control vs. CRC	qRT-PCR	Zekri et al. [108]
miR-223	diagnostic	up-regulation	tissues, stool, plasma	*n* = 907	control vs. CRC	qRT-PCR	Chang et al. [113]
miR-224	diagnostic	down-regulation	stool	*n* = 131	control vs. CRC	qRT-PCR	Zhu et al. [93]
miR-224	prognostic	up-regulation	tissues	*n* = 108	shorter DFS	qRT-PCR	Zhang et al. [94]
miR-224	prognostic	up-regulation	tissues	*n* = 621	shorter OS	miRNA microarrays, qRT-PCR	Ling et al. [95]
miR-224	prognostic	up-regulation	tissues	*n* = 110	shorter OS	qRT-PCR	Liao et al. [97]
miR-375	diagnostic, prognostic	down-regulation	serum	*n* = 91	control vs. CRC, TNM stages, poor prognosis	qRT-PCR	Elshafei et al. [112]
miR-378	diagnostic	up-regulation	plasma	*n* = 65	control vs. CRC	qRT-PCR	Zanutto et al. [103]
miR-378	prognostic	down-regulation	tissues	*n* = 84	shorter OS	qRT-PCR	Zhang et al. [105]
miR-378a-3p	prognostic	down-regulation	tissues	*n* = 96	shorter OS	qRT-PCR	Li et al. [107]
miR-378a-5p	prognostic	down-regulation	tissues	*n* = 96	shorter OS	qRT-PCR	Li et al. [107]
miR-506	diagnostic	up-regulation	plasma	*n* = 126	control vs. CRC	qRT-PCR	Krawczyk et al. [128]
miR-625-3p	diagnostic	down-regulation	tissues	*n* = 54	control vs. CRC	qRT-PCR	Baltruskeviciene et al. [121]
miR-664-3p	prognostic	down-regulation	tissues	*n* = 63	shorter OS, shorter PFS	qRT-PCR	Fiala et al. [118]
miR-760	diagnostic, prognostic	down-regulation	serum	*n* = 91	control vs. CRC, TNM stages, poor prognosis	qRT-PCR	Elshafei et al. [112]
miR-1290	prognostic	up-regulation	tissues	*n* = 291	shorter OS, shorter DFS	miRNA microarrays, qRT-PCR	Ye et al. [129]
miR-4316	diagnostic	up-regulation	plasma	*n* = 126	control vs. CRC	qRT-PCR	Krawczyk et al. [128]

RFS—relapse-free survival.

**Table 3:** The significance of miRNA panels as diagnostic and prognostic biomarkers of colorectal cancer.

MiRNA Panel	Biomarker Type	Regulation in CRC	Source of miRNA	Cohort Size	Correlation/Differentiation	Detection Method	Authors
miR-15b miR-17 miR-21 miR-26b miR-145	diagnostic	up-regulation	plasma	*n* = 280	control vs. CRC	qRT-PCR	Pan et al. [133]
miR-1246 miR-202-3p miR-21-3p miR-1229-3p miR-532-3p	diagnostic	up-regulation (miR-1246, miR-1229-3p, miR-532-3p) down-regulation (miR-202-3p, miR-21-3p)	serum	*n* = 575	control vs. CRC vs. colorectal adenomas	qRT-PCR	Guo et al. [134]
miR-19a-3p miR-21-5p miR-425-5p	diagnostic	up-regulation	serum	*n* = 334	control vs. CRC	qRT-PCR	Zhu et al. [135]
miR-409-3p miR-7 miR-93	diagnostic	up-regulation (miR-409-3p) down-regulation (miR-7, miR-93)	plasma	*n* = 241	control vs. CRC	miRNA microarrays, qRT-PCR	Wang et al. [136]
miR-431 miR-15b miR-139-3p	diagnostic	up-regulation	plasma	*n* = 87	control vs. stage IV CRC	microfluidic array technology, qRT-PCR	Kanaan et al. [137]
miR-431 miR-139-3p	diagnostic	up-regulation	plasma	*n* = 87	control vs. CRC	microfluidic array technology, qRT-PCR	Kanaan et al. [137]
miR-331 miR-15b miR-21 miR-142-3p miR-339-3p	diagnostic	up-regulation	plasma	*n* = 87	colorectal adenomas vs. CRC	microfluidic array technology, qRT-PCR	Kanaan et al. [137]
miR-18a miR-21 miR-22 miR-25	diagnostic	up-regulation (miR-18a, miR-21, miR-25) down-regulation (miR-22)	plasma	*n* = 201	control vs. CRC	semi-quantitative RT-PCR	Wikberg et al. [138]
miR-7 miR-93 miR-195 miR-141 miR-494 let-7b	prognostic	up-regulation (miR-7, miR-141, miR-494) down-regulation (miR-93, miR-195, let-7b))	tissues	*n* = 104	non-early relapsed CRC vs. early relapsed CRC	qRT-PCR	Yang et al. [139]
miR-21 miR-15b miR-29a miR-92a miR-125b miR-223	diagnostic	up-regulation	serum	*n* = 163	control vs. CRC	qRT-PCR	Liu et al. [140]

## Abbreviations

3′-UTR	3′-untranslated region
5-FU	5-fluorouracil
AJCC	American Joint Committee on Cancer
AUC	area under the curve
BIC	MIR155 host gene
CDK6	cyclin-dependent kinase 6
CEA	carcinoembryonic antigen
CRC	colorectal cancer
DCBE	double-contrast barium enema
DFS	disease-free survival
FISH	fluorescence in situ hybrydization
FOBT	fecal occult blood test
HCV	hepatitis C virus
ISH	in situ hybridization
miRNAs	microRNAs
mRNA	messenger RNA
NGS	next-generation sequencing
OS	overall survival
PFS	progression-free survival
PPV	positive predictive value
PTEN	phosphatase and tensin homolog
RFS	relapse-free survival
qRT-PCR	quantitative real-time reverse transcriptase polymerase chain reaction
ROC	receiver operating characteristic
ROCK1	rho-associated protein kinase 1
TGF-β	transforming growth factor β
TNM	tumor-node-metastasis
UICC	Union for International Cancer Control
VEGF	vascular endothelial growth factor
ZEB-1	zinc finger E-box binding homeobox 1 protein

## References

[1] Global Cancer Observatory. http://gco.iarc.fr/today/home.

[2] Maida M., Macaluso F.S., Ianiro G., Mangiola F., Sinagra E., Hold G., Maida C., Cammarota G., Gasbarrini A., Scarpulla G. (2017). Screening of colorectal cancer: Present and future. Expert Rev. Anticancer Ther..

[3] Wolpin B.M., Mayer R.J. (2008). Systemic treatment of colorectal cancer. Gastroenterology.

[4] Tétreault N., De Guire V. (2013). miRNAs: Their discovery, biogenesis and mechanism of action. Clin. Biochem..

[5] Lewis B.P., Burge C.B., Bartel D.P. (2005). Conserved seed pairing, often flanked by adenosines, indicates that thousands of human genes are microRNA targets. Cell.

[6] Friedman R.C., Farh K.K.-H., Burge C.B., Bartel D.P. (2009). Most mammalian mRNAs are conserved targets of microRNAs. Genome Res..

[7] Volinia S., Calin G.A., Liu C.-G., Ambs S., Cimmino A., Petrocca F., Visone R., Iorio M., Roldo C., Ferracin M. (2006). A microRNA expression signature of human solid tumors defines cancer gene targets. Proc. Natl. Acad. Sci. USA.

[8] Lu J., Getz G., Miska E.A., Alvarez-Saavedra E., Lamb J., Peck D., Sweet-Cordero A., Ebert B.L., Mak R.H., Ferrando A.A. (2005). MicroRNA expression profiles classify human cancers. Nature.

[9] Tan W., Liu B., Qu S., Liang G., Luo W., Gong C. (2018). MicroRNAs and cancer: Key paradigms in molecular therapy. Oncol. Lett..

[10] Chaffer C.L., Weinberg R.A. (2011). A perspective on cancer cell metastasis. Science.

[11] Ahlquist D.A. (2010). Molecular detection of colorectal neoplasia. Gastroenterology.

[12] Muniyappa M.K., Dowling P., Henry M., Meleady P., Doolan P., Gammell P., Clynes M., Barron N. (2009). MiRNA-29a regulates the expression of numerous proteins and reduces the invasiveness and proliferation of human carcinoma cell lines. Eur. J. Cancer.

[13] Eminaga S., Christodoulou D.C., Vigneault F., Church G.M., Seidman J.G. (2013). Quantification of microRNA expression with next-generation sequencing. Curr. Protoc. Mol. Biol..

[14] Slaby O., Svoboda M., Fabian P., Smerdova T., Knoflickova D., Bednarikova M., Nenutil R., Vyzula R. (2007). Altered expression of miR-21, miR-31, miR-143 and miR-145 is related to clinicopathologic features of colorectal cancer. Oncology.

[15] Pfeffer U., Romeo F., Noonan D.M., Albini A. (2009). Prediction of breast cancer metastasis by genomic profiling: Where do we stand?. Clin. Exp. Metastasis.

[16] Schetter A.J., Leung S.Y., Sohn J.J., Zanetti K.A., Bowman E.D., Yanaihara N., Yuen S.T., Chan T.L., Kwong D.L.W., Au G.K.H. (2008). MicroRNA expression profiles associated with prognosis and therapeutic outcome in colon adenocarcinoma. JAMA.

[17] Wang C.-J., Zhou Z.-G., Wang L., Yang L., Zhou B., Gu J., Chen H.-Y., Sun X.-F. (2009). Clinicopathological significance of microRNA-31, -143 and -145 expression in colorectal cancer. Dis. Markers.

[18] Koga Y., Yasunaga M., Takahashi A., Kuroda J., Moriya Y., Akasu T., Fujita S., Yamamoto S., Baba H., Matsumura Y. (2010). MicroRNA expression profiling of exfoliated colonocytes isolated from feces for colorectal cancer screening. Cancer Prev. Res..

[19] Kulda V., Pesta M., Topolcan O., Liska V., Treska V., Sutnar A., Rupert K., Ludvikova M., Babuska V., Holubec L. (2010). Relevance of miR-21 and miR-143 expression in tissue samples of colorectal carcinoma and its liver metastases. Cancer Genet. Cytogenet..

[20] Sundaram P., Hultine S., Smith L.M., Dews M., Fox J.L., Biyashev D., Schelter J.M., Huang Q., Cleary M.A., Volpert O.V. (2011). p53-responsive miR-194 inhibits thrombospondin-1 and promotes angiogenesis in colon cancers. Cancer Res..

[21] Wang X., Wang J., Ma H., Zhang J., Zhou X. (2012). Downregulation of miR-195 correlates with lymph node metastasis and poor prognosis in colorectal cancer. Med. Oncol..

[22] Wu X., Somlo G., Yu Y., Palomares M.R., Li A.X., Zhou W., Chow A., Yen Y., Rossi J.J., Gao H. (2012). De novo sequencing of circulating miRNAs identifies novel markers predicting clinical outcome of locally advanced breast cancer. J. Transl. Med..

[23] Oue N., Anami K., Schetter A.J., Moehler M., Okayama H., Khan M.A., Bowman E.D., Mueller A., Schad A., Shimomura M. (2014). High miR-21 expression from FFPE tissues is associated with poor survival and response to adjuvant chemotherapy in colon cancer. Int. J. Cancer.

[24] Zhao G., Zhang J., Shi Y., Qin Q., Liu Y., Wang B., Tian K., Deng S., Li X., Zhu S. (2013). MiR-130b is a prognostic marker and inhibits cell proliferation and invasion in pancreatic cancer through targeting STAT3. PLoS ONE.

[25] Niyazi M., Zehentmayr F., Niemöller O.M., Eigenbrod S., Kretzschmar H., Schulze-Osthoff K., Tonn J.-C., Atkinson M., Mörtl S., Belka C. (2011). MiRNA expression patterns predict survival in glioblastoma. Radiat. Oncol..

[26] Krützfeldt J., Rajewsky N., Braich R., Rajeev K.G., Tuschl T., Manoharan M., Stoffel M. (2005). Silencing of microRNAs in vivo with “antagomirs”. Nature.

[27] Lanford R.E., Hildebrandt-Eriksen E.S., Petri A., Persson R., Lindow M., Munk M.E., Kauppinen S., Ørum H. (2010). Therapeutic silencing of microRNA-122 in primates with chronic hepatitis C virus infection. Science.

[28] Janssen H.L.A., Reesink H.W., Lawitz E.J., Zeuzem S., Rodriguez-Torres M., Patel K., van der Meer A.J., Patick A.K., Chen A., Zhou Y. (2013). Treatment of HCV infection by targeting microRNA. N. Engl. J. Med..

[29] Young D.D., Connelly C.M., Grohmann C., Deiters A. (2010). Small molecule modifiers of microRNA miR-122 function for the treatment of hepatitis C virus infection and hepatocellular carcinoma. J. Am. Chem. Soc..

[30] Shi C., Yang Y., Xia Y., Okugawa Y., Yang J., Liang Y., Chen H., Zhang P., Wang F., Han H. (2016). Novel evidence for an oncogenic role of microRNA-21 in colitis-associated colorectal cancer. Gut.

[31] Jiang L., Hermeking H. (2017). miR-34a and miR-34b/c Suppress Intestinal Tumorigenesis. Cancer Res..

[32] Rokavec M., Öner M.G., Li H., Jackstadt R., Jiang L., Lodygin D., Kaller M., Horst D., Ziegler P.K., Schwitalla S. (2014). IL-6R/STAT3/miR-34a feedback loop promotes EMT-mediated colorectal cancer invasion and metastasis. J. Clin. Investig..

[33] Toiyama Y., Takahashi M., Hur K., Nagasaka T., Tanaka K., Inoue Y., Kusunoki M., Boland C.R., Goel A. (2013). Serum miR-21 as a diagnostic and prognostic biomarker in colorectal cancer. J. Natl. Cancer Inst..

[34] Wu Y., Song Y., Xiong Y., Wang X., Xu K., Han B., Bai Y., Li L., Zhang Y., Zhou L. (2017). MicroRNA-21 (Mir-21) Promotes Cell Growth and Invasion by Repressing Tumor Suppressor PTEN in Colorectal Cancer. Cell. Physiol. Biochem..

[35] Nana-Sinkam S.P., Fabbri M., Croce C.M. (2010). MicroRNAs in cancer: Personalizing diagnosis and therapy. Ann. N. Y. Acad. Sci..

[36] Yamamichi N., Shimomura R., Inada K., Sakurai K., Haraguchi T., Ozaki Y., Fujita S., Mizutani T., Furukawa C., Fujishiro M. (2009). Locked nucleic acid in situ hybridization analysis of miR-21 expression during colorectal cancer development. Clin. Cancer Res..

[37] Bastaminejad S., Taherikalani M., Ghanbari R., Akbari A., Shabab N., Saidijam M. (2017). Investigation of MicroRNA-21 Expression Levels in Serum and Stool as a Potential Non-Invasive Biomarker for Diagnosis of Colorectal Cancer. Iran. Biomed. J..

[38] Wu C.W., Ng S.S.M., Dong Y.J., Ng S.C., Leung W.W., Lee C.W., Wong Y.N., Chan F.K.L., Yu J., Sung J.J.Y. (2012). Detection of miR-92a and miR-21 in stool samples as potential screening biomarkers for colorectal cancer and polyps. Gut.

[39] Link A., Balaguer F., Shen Y., Nagasaka T., Lozano J.J., Boland C.R., Goel A. (2010). Fecal MicroRNAs as novel biomarkers for colon cancer screening. Cancer Epidemiol. Biomark. Prev..

[40] Fukushima Y., Iinuma H., Tsukamoto M., Matsuda K., Hashiguchi Y. (2015). Clinical significance of microRNA-21 as a biomarker in each Dukes’ stage of colorectal cancer. Oncol. Rep..

[41] Kjaer-Frifeldt S., Hansen T.F., Nielsen B.S., Joergensen S., Lindebjerg J., Soerensen F.B., dePont Christensen R., Jakobsen A., Danish Colorectal Cancer Group (2012). The prognostic importance of miR-21 in stage II colon cancer: A population-based study. Br. J. Cancer.

[42] Nielsen B.S., Jørgensen S., Fog J.U., Søkilde R., Christensen I.J., Hansen U., Brünner N., Baker A., Møller S., Nielsen H.J. (2011). High levels of microRNA-21 in the stroma of colorectal cancers predict short disease-free survival in stage II colon cancer patients. Clin. Exp. Metastasis.

[43] Shibuya H., Iinuma H., Shimada R., Horiuchi A., Watanabe T. (2010). Clinicopathological and prognostic value of microRNA-21 and microRNA-155 in colorectal cancer. Oncology.

[44] Xia X., Yang B., Zhai X., Liu X., Shen K., Wu Z., Cai J. (2013). Prognostic role of microRNA-21 in colorectal cancer: A meta-analysis. PLoS ONE.

[45] Chen Z., Liu H., Jin W., Ding Z., Zheng S., Yu Y. (2016). Tissue microRNA-21 expression predicted recurrence and poor survival in patients with colorectal cancer—A meta-analysis. Oncotargets Ther..

[46] Peng Q., Zhang X., Min M., Zou L., Shen P., Zhu Y. (2017). The clinical role of microRNA-21 as a promising biomarker in the diagnosis and prognosis of colorectal cancer: A systematic review and meta-analysis. Oncotarget.

[47] Kang W.K., Lee J.K., Oh S.T., Lee S.H., Jung C.K. (2015). Stromal expression of miR-21 in T3-4a colorectal cancer is an independent predictor of early tumor relapse. BMC Gastroenterol..

[48] Kanaan Z., Rai S.N., Eichenberger M.R., Roberts H., Keskey B., Pan J., Galandiuk S. (2012). Plasma miR-21: A potential diagnostic marker of colorectal cancer. Ann. Surg..

[49] Tsukamoto M., Iinuma H., Yagi T., Matsuda K., Hashiguchi Y. (2017). Circulating Exosomal MicroRNA-21 as a Biomarker in Each Tumor Stage of Colorectal Cancer. Oncology.

[50] Ahmed F.E., Ahmed N.C., Vos P.W., Bonnerup C., Atkins J.N., Casey M., Nuovo G.J., Naziri W., Wiley J.E., Mota H. (2013). Diagnostic microRNA markers to screen for sporadic human colon cancer in stool: I. Proof of principle. Cancer Genom. Proteom..

[51] Wang Y., Zhang X., Li H., Yu J., Ren X. (2013). The role of miRNA-29 family in cancer. Eur. J. Cell Biol..

[52] Wang L.-G., Gu J. (2012). Serum microRNA-29a is a promising novel marker for early detection of colorectal liver metastasis. Cancer Epidemiol..

[53] Huang Z., Huang D., Ni S., Peng Z., Sheng W., Du X. (2010). Plasma microRNAs are promising novel biomarkers for early detection of colorectal cancer. Int. J. Cancer.

[54] Al-Sheikh Y.A., Ghneim H.K., Softa K.I., Al-Jobran A.A., Al-Obeed O., Mohamed M.A.V., Abdulla M., Aboul-Soud M.A.M. (2016). Expression profiling of selected microRNA signatures in plasma and tissues of Saudi colorectal cancer patients by qPCR. Oncol. Lett..

[55] Li L., Guo Y., Chen Y., Wang J., Zhen L., Guo X., Liu J., Jing C. (2016). The Diagnostic Efficacy and Biological Effects of microRNA-29b for Colon Cancer. Technol. Cancer Res. Treat..

[56] Basati G., Razavi A.E., Pakzad I., Malayeri F.A. (2016). Circulating levels of the miRNAs, miR-194, and miR-29b, as clinically useful biomarkers for colorectal cancer. Tumour Biol..

[57] Tang W., Zhu Y., Gao J., Fu J., Liu C., Liu Y., Song C., Zhu S., Leng Y., Wang G. (2014). MicroRNA-29a promotes colorectal cancer metastasis by regulating matrix metalloproteinase 2 and E-cadherin via KLF4. Br. J. Cancer.

[58] Weissmann-Brenner A., Kushnir M., Lithwick Yanai G., Aharonov R., Gibori H., Purim O., Kundel Y., Morgenstern S., Halperin M., Niv Y. (2012). Tumor microRNA-29a expression and the risk of recurrence in stage II colon cancer. Int. J. Oncol..

[59] Kuo T.-Y., Hsi E., Yang I.-P., Tsai P.-C., Wang J.-Y., Juo S.-H.H. (2012). Computational analysis of mRNA expression profiles identifies microRNA-29a/c as predictor of colorectal cancer early recurrence. PLoS ONE.

[60] Inoue A., Yamamoto H., Uemura M., Nishimura J., Hata T., Takemasa I., Ikenaga M., Ikeda M., Murata K., Mizushima T. (2015). MicroRNA-29b is a Novel Prognostic Marker in Colorectal Cancer. Ann. Surg. Oncol..

[61] Yuan L., Zhou C., Lu Y., Hong M., Zhang Z., Zhang Z., Chang Y., Zhang C., Li X. (2015). IFN-γ-mediated IRF1/miR-29b feedback loop suppresses colorectal cancer cell growth and metastasis by repressing IGF1. Cancer Lett..

[62] Ulivi P., Canale M., Passardi A., Marisi G., Valgiusti M., Frassineti G.L., Calistri D., Amadori D., Scarpi E. (2018). Circulating Plasma Levels of miR-20b, miR-29b and miR-155 as Predictors of Bevacizumab Efficacy in Patients with Metastatic Colorectal Cancer. Int. J. Mol. Sci..

[63] Misso G., Di Martino M.T., De Rosa G., Farooqi A.A., Lombardi A., Campani V., Zarone M.R., Gullà A., Tagliaferri P., Tassone P. (2014). Mir-34: A new weapon against cancer?. Mol. Ther. Nucleic Acids.

[64] Bu P., Chen K.-Y., Chen J.H., Wang L., Walters J., Shin Y.J., Goerger J.P., Sun J., Witherspoon M., Rakhilin N. (2013). A microRNA miR-34a-regulated bimodal switch targets Notch in colon cancer stem cells. Cell Stem Cell.

[65] Wu X., Song Y.-C., Cao P.-L., Zhang H., Guo Q., Yan R., Diao D.-M., Cheng Y., Dang C.-X. (2014). Detection of miR-34a and miR-34b/c in stool sample as potential screening biomarkers for noninvasive diagnosis of colorectal cancer. Med. Oncol..

[66] Aherne S.T., Madden S.F., Hughes D.J., Pardini B., Naccarati A., Levy M., Vodicka P., Neary P., Dowling P., Clynes M. (2015). Circulating miRNAs miR-34a and miR-150 associated with colorectal cancer progression. BMC Cancer.

[67] Gao J., Li N., Dong Y., Li S., Xu L., Li X., Li Y., Li Z., Ng S.S., Sung J.J. (2015). miR-34a-5p suppresses colorectal cancer metastasis and predicts recurrence in patients with stage II/III colorectal cancer. Oncogene.

[68] Ma Y., Bao-Han W., Lv X., Su Y., Zhao X., Yin Y., Zhang X., Zhou Z., MacNaughton W.K., Wang H. (2013). MicroRNA-34a mediates the autocrine signaling of PAR2-activating proteinase and its role in colonic cancer cell proliferation. PLoS ONE.

[69] Siemens H., Neumann J., Jackstadt R., Mansmann U., Horst D., Kirchner T., Hermeking H. (2013). Detection of miR-34a promoter methylation in combination with elevated expression of c-Met and β-catenin predicts distant metastasis of colon cancer. Clin. Cancer Res..

[70] Tazawa H., Tsuchiya N., Izumiya M., Nakagama H. (2007). Tumor-suppressive miR-34a induces senescence-like growth arrest through modulation of the E2F pathway in human colon cancer cells. Proc. Natl. Acad. Sci. USA.

[71] de Almeida A.L.N.R., Bernardes M.V.A.A., Feitosa M.R., Peria F.M., da Tirapelli D.P.C., da Rocha J.J.R., Feres O. (2016). Serological under expression of microRNA-21, microRNA-34a and microRNA-126 in colorectal cancer. Acta Cir. Bras..

[72] Li C., Wang Y., Lu S., Zhang Z., Meng H., Liang L., Zhang Y., Song B. (2015). MiR-34a inhibits colon cancer proliferation and metastasis by inhibiting platelet-derived growth factor receptor α. Mol. Med. Rep..

[73] Zhang X., Ai F., Li X., Tian L., Wang X., Shen S., Liu F. (2017). MicroRNA-34a suppresses colorectal cancer metastasis by regulating Notch signaling. Oncol. Lett..

[74] Hiyoshi Y., Schetter A.J., Okayama H., Inamura K., Anami K., Nguyen G.H., Horikawa I., Hawkes J.E., Bowman E.D., Leung S.Y. (2015). Increased microRNA-34b and -34c predominantly expressed in stromal tissues is associated with poor prognosis in human colon cancer. PLoS ONE.

[75] Akao Y., Noguchi S., Iio A., Kojima K., Takagi T., Naoe T. (2011). Dysregulation of microRNA-34a expression causes drug-resistance to 5-FU in human colon cancer DLD-1 cells. Cancer Lett..

[76] Sun C., Wang F.-J., Zhang H.-G., Xu X.-Z., Jia R.-C., Yao L., Qiao P.-F. (2017). miR-34a mediates oxaliplatin resistance of colorectal cancer cells by inhibiting macroautophagy via transforming growth factor-β/Smad4 pathway. World J. Gastroenterol..

[77] Xi Z.-W., Xin S.-Y., Zhou L.-Q., Yuan H.-X., Wang Q., Chen K.-X. (2015). Downregulation of rho-associated protein kinase 1 by miR-124 in colorectal cancer. World J. Gastroenterol..

[78] Feng T., Xu D., Tu C., Li W., Ning Y., Ding J., Wang S., Yuan L., Xu N., Qian K. (2015). MiR-124 inhibits cell proliferation in breast cancer through downregulation of CDK4. Tumour Biol..

[79] Harada T., Yamamoto E., Yamano H., Nojima M., Maruyama R., Kumegawa K., Ashida M., Yoshikawa K., Kimura T., Harada E. (2014). Analysis of DNA methylation in bowel lavage fluid for detection of colorectal cancer. Cancer Prev. Res..

[80] Faber C., Kirchner T., Hlubek F. (2009). The impact of microRNAs on colorectal cancer. Virchows Arch. Int. J. Pathol..

[81] Ueda Y., Ando T., Nanjo S., Ushijima T., Sugiyama T. (2014). DNA methylation of microRNA-124a is a potential risk marker of colitis-associated cancer in patients with ulcerative colitis. Dig. Dis. Sci..

[82] Wang M.-J., Li Y., Wang R., Wang C., Yu Y.-Y., Yang L., Zhang Y., Zhou B., Zhou Z.-G., Sun X.-F. (2013). Downregulation of microRNA-124 is an independent prognostic factor in patients with colorectal cancer. Int. J. Colorectal Dis..

[83] Jinushi T., Shibayama Y., Kinoshita I., Oizumi S., Jinushi M., Aota T., Takahashi T., Horita S., Dosaka-Akita H., Iseki K. (2014). Low expression levels of microRNA-124-5p correlated with poor prognosis in colorectal cancer via targeting of SMC4. Cancer Med..

[84] Slattery M.L., Pellatt A.J., Lee F.Y., Herrick J.S., Samowitz W.S., Stevens J.R., Wolff R.K., Mullany L.E. (2017). Infrequently expressed miRNAs influence survival after diagnosis with colorectal cancer. Oncotarget.

[85] Colangelo T., Fucci A., Votino C., Sabatino L., Pancione M., Laudanna C., Binaschi M., Bigioni M., Maggi C.A., Parente D. (2013). MicroRNA-130b promotes tumor development and is associated with poor prognosis in colorectal cancer. Neoplasia.

[86] Chen W., Tong K., Yu J. (2017). MicroRNA-130a is upregulated in colorectal cancer and promotes cell growth and motility by directly targeting forkhead box F2. Mol. Med. Rep..

[87] Sempere L.F., Preis M., Yezefski T., Ouyang H., Suriawinata A.A., Silahtaroglu A., Conejo-Garcia J.R., Kauppinen S., Wells W., Korc M. (2010). Fluorescence-based codetection with protein markers reveals distinct cellular compartments for altered MicroRNA expression in solid tumors. Clin. Cancer Res..

[88] Lv Z., Fan Y., Chen H., Zhao D. (2015). Investigation of microRNA-155 as a serum diagnostic and prognostic biomarker for colorectal cancer. Tumour Biol..

[89] Hongliang C., Shaojun H., Aihua L., Hua J. (2014). Correlation between expression of miR-155 in colon cancer and serum carcinoembryonic antigen level and its contribution to recurrence and metastasis forecast. Saudi Med. J..

[90] Zhang G.-J., Xiao H.-X., Tian H.-P., Liu Z.-L., Xia S.-S., Zhou T. (2013). Upregulation of microRNA-155 promotes the migration and invasion of colorectal cancer cells through the regulation of claudin-1 expression. Int. J. Mol. Med..

[91] Qu Y.-L., Wang H.-F., Sun Z.-Q., Tang Y., Han X.-N., Yu X.-B., Liu K. (2015). Up-regulated miR-155-5p promotes cell proliferation, invasion and metastasis in colorectal carcinoma. Int. J. Clin. Exp. Pathol..

[92] Wang Y., Lee C.G.L. (2011). Role of miR-224 in hepatocellular carcinoma: A tool for possible therapeutic intervention?. Epigenomics.

[93] Zhu Y., Xu A., Li J., Fu J., Wang G., Yang Y., Cui L., Sun J. (2016). Fecal miR-29a and miR-224 as the noninvasive biomarkers for colorectal cancer. Cancer Biomark. Sect. Dis. Markers.

[94] Zhang G.-J., Zhou H., Xiao H.-X., Li Y., Zhou T. (2013). Up-regulation of miR-224 promotes cancer cell proliferation and invasion and predicts relapse of colorectal cancer. Cancer Cell Int..

[95] Ling H., Pickard K., Ivan C., Isella C., Ikuo M., Mitter R., Spizzo R., Bullock M., Braicu C., Pileczki V. (2016). The clinical and biological significance of MIR-224 expression in colorectal cancer metastasis. Gut.

[96] Wang Z., Yang J., Di J., Cui M., Xing J., Wu F., Wu W., Yang H., Zhang C., Yao Z. (2017). Downregulated USP3 mRNA functions as a competitive endogenous RNA of SMAD4 by sponging miR-224 and promotes metastasis in colorectal cancer. Sci. Rep..

[97] Liao W.-T., Li T.-T., Wang Z.-G., Wang S.-Y., He M.-R., Ye Y.-P., Qi L., Cui Y.-M., Wu P., Jiao H.-L. (2013). microRNA-224 promotes cell proliferation and tumor growth in human colorectal cancer by repressing PHLPP1 and PHLPP2. Clin. Cancer Res..

[98] Ke T.-W., Hsu H.-L., Wu Y.-H., Chen W.T.-L., Cheng Y.-W., Cheng C.-W. (2014). MicroRNA-224 suppresses colorectal cancer cell migration by targeting Cdc42. Dis. Markers.

[99] Zhang L., Huang L.-S., Chen G., Feng Z.-B. (2017). Potential Targets and Clinical Value of MiR-224-5p in Cancers of the Digestive Tract. Cell. Physiol. Biochem..

[100] Mosakhani N., Sarhadi V.K., Borze I., Karjalainen-Lindsberg M.-L., Sundström J., Ristamäki R., Osterlund P., Knuutila S. (2012). MicroRNA profiling differentiates colorectal cancer according to KRAS status. Genes Chromosomes Cancer.

[101] Wang Y.X., Zhang X.Y., Zhang B.F., Yang C.Q., Chen X.M., Gao H.J. (2010). Initial study of microRNA expression profiles of colonic cancer without lymph node metastasis. J. Dig. Dis..

[102] Chen L., Xu S., Xu H., Zhang J., Ning J., Wang S. (2012). MicroRNA-378 is associated with non-small cell lung cancer brain metastasis by promoting cell migration, invasion and tumor angiogenesis. Med. Oncol..

[103] Zanutto S., Pizzamiglio S., Ghilotti M., Bertan C., Ravagnani F., Perrone F., Leo E., Pilotti S., Verderio P., Gariboldi M. (2014). Circulating miR-378 in plasma: A reliable, haemolysis-independent biomarker for colorectal cancer. Br. J. Cancer.

[104] Wang Z., Ma B., Ji X., Deng Y., Zhang T., Zhang X., Gao H., Sun H., Wu H., Chen X. (2015). MicroRNA-378-5p suppresses cell proliferation and induces apoptosis in colorectal cancer cells by targeting BRAF. Cancer Cell Int..

[105] Zhang G.-J., Zhou H., Xiao H.-X., Li Y., Zhou T. (2014). MiR-378 is an independent prognostic factor and inhibits cell growth and invasion in colorectal cancer. BMC Cancer.

[106] Zeng M., Zhu L., Li L., Kang C. (2017). miR-378 suppresses the proliferation, migration and invasion of colon cancer cells by inhibiting SDAD1. Cell. Mol. Biol. Lett..

[107] Li H., Dai S., Zhen T., Shi H., Zhang F., Yang Y., Kang L., Liang Y., Han A. (2014). Clinical and biological significance of miR-378a-3p and miR-378a-5p in colorectal cancer. Eur. J. Cancer.

[108] Zekri A.-R.N., Youssef A.S.E.-D., Lotfy M.M., Gabr R., Ahmed O.S., Nassar A., Hussein N., Omran D., Medhat E., Eid S. (2016). Circulating Serum miRNAs as Diagnostic Markers for Colorectal Cancer. PLoS ONE.

[109] Li B., Li B., Sun H., Zhang H. (2017). The predicted target gene validation, function, and prognosis studies of miRNA-22 in colorectal cancer tissue. Tumour Biol..

[110] Kerimis D., Kontos C.K., Christodoulou S., Papadopoulos I.N., Scorilas A. (2017). Elevated expression of miR-24-3p is a potentially adverse prognostic factor in colorectal adenocarcinoma. Clin. Biochem..

[111] Li Y., Sun Z., Liu B., Shan Y., Zhao L., Jia L. (2017). Tumor-suppressive miR-26a and miR-26b inhibit cell aggressiveness by regulating FUT4 in colorectal cancer. Cell Death Dis..

[112] Elshafei A., Shaker O., Abd El-Motaal O., Salman T. (2017). The expression profiling of serum miR-92a, miR-375, and miR-760 in colorectal cancer: An Egyptian study. Tumour Biol..

[113] Chang P.-Y., Chen C.-C., Chang Y.-S., Tsai W.-S., You J.-F., Lin G.-P., Chen T.-W., Chen J.-S., Chan E.-C. (2016). MicroRNA-223 and microRNA-92a in stool and plasma samples act as complementary biomarkers to increase colorectal cancer detection. Oncotarget.

[114] Fu F., Jiang W., Zhou L., Chen Z. (2018). Circulating Exosomal miR-17-5p and miR-92a-3p Predict Pathologic Stage and Grade of Colorectal Cancer. Transl. Oncol..

[115] He Y., Wang G., Zhang L., Zhai C., Zhang J., Zhao X., Jiang X., Zhao Z. (2017). Biological effects and clinical characteristics of microRNA-106a in human colorectal cancer. Oncol. Lett..

[116] Maierthaler M., Benner A., Hoffmeister M., Surowy H., Jansen L., Knebel P., Chang-Claude J., Brenner H., Burwinkel B. (2017). Plasma miR-122 and miR-200 family are prognostic markers in colorectal cancer. Int. J. Cancer.

[117] Liang L., Gao C., Li Y., Sun M., Xu J., Li H., Jia L., Zhao Y. (2017). miR-125a-3p/FUT5-FUT6 axis mediates colorectal cancer cell proliferation, migration, invasion and pathological angiogenesis via PI3K-Akt pathway. Cell Death Dis..

[118] Fiala O., Pitule P., Hosek P., Liska V., Sorejs O., Bruha J., Vycital O., Buchler T., Poprach A., Topolcan O. (2017). The association of miR-126-3p, miR-126-5p and miR-664-3p expression profiles with outcomes of patients with metastatic colorectal cancer treated with bevacizumab. Tumour Biol..

[119] Ng L., Wan T.M.-H., Man J.H.-W., Chow A.K.-M., Iyer D., Chen G., Yau T.C.-C., Lo O.S.-H., Foo D.C.-C., Poon J.T.-C. (2017). Identification of serum miR-139-3p as a non-invasive biomarker for colorectal cancer. Oncotarget.

[120] Miyoshi J., Toden S., Yoshida K., Toiyama Y., Alberts S.R., Kusunoki M., Sinicrope F.A., Goel A. (2017). MiR-139-5p as a novel serum biomarker for recurrence and metastasis in colorectal cancer. Sci. Rep..

[121] Baltruskeviciene E., Schveigert D., Stankevicius V., Mickys U., Zvirblis T., Bublevic J., Suziedelis K., Aleknavicius E. (2017). Down-regulation of miRNA-148a and miRNA-625-3p in colorectal cancer is associated with tumor budding. BMC Cancer.

[122] Gu X., Jin R., Mao X., Wang J., Yuan J., Zhao G. (2018). Prognostic value of miRNA-181a/b in colorectal cancer: A meta-analysis. Biomark. Med..

[123] Yamazaki N., Koga Y., Taniguchi H., Kojima M., Kanemitsu Y., Saito N., Matsumura Y. (2017). High expression of miR-181c as a predictive marker of recurrence in stage II colorectal cancer. Oncotarget.

[124] Guo X., Zhu Y., Hong X., Zhang M., Qiu X., Wang Z., Qi Z., Hong X. (2017). miR-181d and c-myc-mediated inhibition of CRY2 and FBXL3 reprograms metabolism in colorectal cancer. Cell Death Dis..

[125] Lin M., Duan B., Hu J., Yu H., Sheng H., Gao H., Huang J. (2017). Decreased expression of miR-193a-3p is associated with poor prognosis in colorectal cancer. Oncol. Lett..

[126] Li W., Chang J., Tong D., Peng J., Huang D., Guo W., Zhang W., Li J. (2017). Differential microRNA expression profiling in primary tumors and matched liver metastasis of patients with colorectal cancer. Oncotarget.

[127] Tayel S.I., Fouda E.A.M., Gohar S.F., Elshayeb E.I., El-Sayed E.H., El-Kousy S.M. (2018). Potential role of MicroRNA 200c gene expression in assessment of colorectal cancer. Arch. Biochem. Biophys..

[128] Krawczyk P., Powrózek T., Olesiński T., Dmitruk A., Dziwota J., Kowalski D., Milanowski J. (2017). Evaluation of miR-506 and miR-4316 expression in early and non-invasive diagnosis of colorectal cancer. Int. J. Colorectal Dis..

[129] Ye L., Jiang T., Shao H., Zhong L., Wang Z., Liu Y., Tang H., Qin B., Zhang X., Fan J. (2017). miR-1290 Is a Biomarker in DNA-Mismatch-Repair-Deficient Colon Cancer and Promotes Resistance to 5-Fluorouracil by Directly Targeting hMSH2. Mol. Ther. Nucleic Acids.

[130] Kiss I., Mlčochová J., Součková K., Fabian P., Poprach A., Halamkova J., Svoboda M., Vyzula R., Slaby O. (2017). MicroRNAs as outcome predictors in patients with metastatic colorectal cancer treated with bevacizumab in combination with FOLFOX. Oncol. Lett..

[131] Chang J., Huang L., Cao Q., Liu F. (2016). Identification of colorectal cancer-restricted microRNAs and their target genes based on high-throughput sequencing data. Oncotargets Ther..

[132] Koduru S.V., Tiwari A.K., Hazard S.W., Mahajan M., Ravnic D.J. (2017). Exploration of small RNA-seq data for small non-coding RNAs in Human Colorectal Cancer. J. Genom..

[133] Pan C., Yan X., Li H., Huang L., Yin M., Yang Y., Gao R., Hong L., Ma Y., Shi C. (2017). Systematic literature review and clinical validation of circulating microRNAs as diagnostic biomarkers for colorectal cancer. Oncotarget.

[134] Guo S., Zhang J., Wang B., Zhang B., Wang X., Huang L., Liu H., Jia B. (2018). A 5-serum miRNA panel for the early detection of colorectal cancer. Oncotargets Ther..

[135] Zhu M., Huang Z., Zhu D., Zhou X., Shan X., Qi L.-W., Wu L., Cheng W., Zhu J., Zhang L. (2017). A panel of microRNA signature in serum for colorectal cancer diagnosis. Oncotarget.

[136] Wang S., Xiang J., Li Z., Lu S., Hu J., Gao X., Yu L., Wang L., Wang J., Wu Y. (2015). A plasma microRNA panel for early detection of colorectal cancer. Int. J. Cancer.

[137] Kanaan Z., Roberts H., Eichenberger M.R., Billeter A., Ocheretner G., Pan J., Rai S.N., Jorden J., Williford A., Galandiuk S. (2013). A plasma microRNA panel for detection of colorectal adenomas: A step toward more precise screening for colorectal cancer. Ann. Surg..

[138] Wikberg M.L., Myte R., Palmqvist R., van Guelpen B., Ljuslinder I. (2018). Plasma miRNA can detect colorectal cancer, but how early?. Cancer Med..

[139] Yang I.-P., Tsai H.-L., Miao Z.-F., Huang C.-W., Kuo C.-H., Wu J.-Y., Wang W.-M., Juo S.-H.H., Wang J.-Y. (2016). Development of a deregulating microRNA panel for the detection of early relapse in postoperative colorectal cancer patients. J. Transl. Med..

[140] Liu H.-N., Liu T.-T., Wu H., Chen Y.-J., Tseng Y.-J., Yao C., Weng S.-Q., Dong L., Shen X.-Z. (2018). Serum microRNA signatures and metabolomics have high diagnostic value in colorectal cancer using two novel methods. Cancer Sci..

[141] Wong C.K.W., Fedorak R.N., Prosser C.I., Stewart M.E., van Zanten S.V., Sadowski D.C. (2012). The sensitivity and specificity of guaiac and immunochemical fecal occult blood tests for the detection of advanced colonic adenomas and cancer. Int. J. Colorectal Dis..

[142] Barzi A., Lenz H.-J., Quinn D.I., Sadeghi S. (2017). Comparative effectiveness of screening strategies for colorectal cancer. Cancer.

